# Components and Delivery Formats of Cognitive Behavioral Therapy for Chronic Fatigue Syndrome/Myalgic Encephalomyelitis: A Systematic Review and Component Network Meta‐Analysis

**DOI:** 10.1002/brb3.71513

**Published:** 2026-05-31

**Authors:** Shoujian Wang, Jun Ren, Sitong Fang, Qiukui Hao, Yu‐Qing Zhang, Lingjun Kong, Min Fang

**Affiliations:** ^1^ Department of Tuina Shuguang Hospital Shanghai University of Traditional Chinese Medicine Shanghai China; ^2^ School of Rehabilitation Science McMaster University Hamilton Canada; ^3^ School of Dental Medicine University of Pennsylvania Philadelphia Pennsylvania USA; ^4^ Department of Health Research Methods, Evidence and Impact McMaster University Hamilton Canada; ^5^ CEBIM (Center for Evidence‐Based Integrative Medicine) Guang'anmen Hospital China Academy of Chinese Medical Sciences Beijing China; ^6^ Institute of Tuina, Shanghai Institute of Traditional Chinese Medicine Shanghai China

**Keywords:** chronic fatigue syndrome, cognitive behavioral therapy, component network meta‐analysis

## Abstract

**Objective:**

Chronic fatigue syndrome/myalgic encephalomyelitis (CFS/ME) is a debilitating condition characterized by persistent fatigue, impaired functioning, and substantial societal burden. Although cognitive behavioral therapy (CBT) is commonly used for CFS/ME, the effective components and delivery formats remain unclear.

**Methods:**

We systematically searched MEDLINE, Embase, PsyclNFO, Web of Science, the Cochrane Library, and major English and Chinese databases from inception to January 15, 2025, without language restrictions. Ultimately, a frequentist network meta‐analysis (NMA) and component network meta‐analysis (cNMA) of 16 randomized controlled trials were conducted to assess the associations between CBT components, delivery formats, and patient‐important outcomes.

**Results:**

At the treatment level, guided self‐help CBT (mean difference [MD], −6.89; 95% CI, −9.56 to −4.22; moderate certainty) and individual CBT (MD, −4.81; 95% CI, −6.98 to −2.65; moderate certainty) probably reduced fatigue when measured immediately after treatment. At the component level, goal setting (incremental mean difference [iMD], −8.83; 95% CI, −16.92 to −0.74), third‐wave components (iMD, −6.02; 95% CI, −11.33 to −0.72), and cognitive restructuring (iMD, −4.41; 95% CI, −8.83 to 0.01) were associated with reduced fatigue when measured immediately after treatment. Psychoeducation was potentially counterproductive (iMD, 5.23; 95% CI, 0.64 to 9.82). At the end of follow‐up, cognitive restructuring was associated with reduced fatigue and depression, and improved physical function; goal setting was also associated with improved physical function. The combination of goal setting, third‐wave components, and cognitive restructuring delivered via guided self‐help may be an efficacious component‐based intervention relative to the nonspecific treatment component (iMD, −16.33; 95% CI, −26.68 to −5.99).

**Conclusions:**

The effective CBT combination probably involves goal setting, third‐wave components, and cognitive restructuring delivered via guided self‐help, while psychoeducation may be detrimental. Future studies should prospectively examine core CBT components and their interactions, as well as incorporate measures of dose and intensity to guide more personalized and scalable interventions.

**Trial Registration:**

CRD420251018083

## Graphical abstract

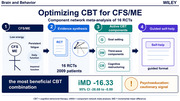

## 1 Introduction

Chronic fatigue syndrome/myalgic encephalomyelitis (CFS/ME) is a long‐term, debilitating condition characterized by persistent fatigue lasting for 6 months or more, along with mood disturbances, and physical impairments (Balshem et al. 2011; Bested and Marshall 2015). The global prevalence of CFS/ME is estimated at approximately 1%, with a higher incidence in females (Lim et al. 2020). However, due to variations in diagnostic criteria, the actual number of individuals affected by CFS/ME may be significantly higher than reported (Lim et al. 2020). Most patients with CFS/ME struggle with routine social, educational, and occupational activities, leading to considerable economic burdens on both individuals and society (Castro‐Marrero et al. 2019; Rimbaut et al. 2016; Zhao et al. 2023).

Cognitive behavioral therapy (CBT), a structured psychological intervention, has emerged as one of the most studied and widely recommended treatments for CFS/ME (Nijhof et al. 2012; Sharpe et al. 2015; White et al. 2011). It includes a wide array of cognitive and behavioral skills such as cognitive restructuring, behavioral activation, and goal setting. The training of these skills is usually flexible. Healthcare providers and researchers have proposed various delivery formats for CBT, including group, individual, telephone, digital assisted, and self‐help formats. Previous reviews have shown that CBT for CFS/ME effectively reduces fatigue, improves physical function, and alleviates depression and anxiety (Kuut et al. 2024a; Maas Genannt Bermpohl et al. 2024; Price and Couper 2000). However, the specific roles of each individual component remain unclear, and the optimal combination of treatment components and delivery formats for CFS/ME is not well understood.

Traditional methods for examining treatment components often rely on dismantling studies, where a complete treatment package is compared to one that excludes a specific component (Bell et al. 2013). These studies are typically underpowered and suffer from methodological limitations. Furthermore, the heterogeneity of components studied and the diverse conditions addressed have made it challenging to synthesize findings across studies (Cuijpers et al. 2019). Recent advancements in evidence synthesis have enabled the dismantling of complex interventions through component network meta‐analysis (cNMA). This extension of standard NMA estimates the individual efficacy of each component within a network of randomized controlled trials (RCTs) (Efthimiou et al. 2016; López‐López et al. 2019; Petropoulou et al. 2021; Pompoli et al. 2018; Welton et al. 2009). cNMA enhances statistical power by integrating both direct and indirect comparisons, while maintaining the integrity of the randomized structure of the evidence. Treatment effects are first estimated separately within each study, and then these study‐specific estimates are pooled across the network (Welton et al. 2009).

In this study, we explored the effective delivery formats and treatment components of CBT for CFS/ME using cNMA, aiming to identify the most beneficial combination of components.

## 2 Methods

The protocol was prospectively registered in PROSPERO (CRD420251018083), and we reported the results following the Preferred Reporting Items for Systematic reviews and Meta‐Analyses (PRISMA) 2020 and the extension statement for NMA (Hutton et al. 2015; Page et al. 2021).

### 2.1 Search Strategy

We searched the following electronic databases from their inception to January 15, 2025: MEDLINE (OVID platform), EMBASE (OVID platform), PsycINFO (OVID platform), Web of Science, Cochrane Library, Chinese BioMedical Literature databases, China Knowledge Resource Integrated Database (CNKI), Weipu Database for Chinese Technical Periodicals (VIP), and Wanfang Data Information (see Appendix S1 for search strategies). We also screened additional eligible studies identified from the reference lists of related systematic reviews and guidelines, without publication status restrictions.

### 2.2 Eligibility Criteria

We included RCTs evaluating CBT for CFS/ME, with no restrictions on sex or age. Eligible participants had to meet established diagnostic criteria for CFS/ME, such as CDC‐1988, CDC‐1994, NICE‐2021, IOM‐2015, or Oxford‐1991. Eligible comparisons involved any delivery format of CBT versus another format of CBT or a control intervention for CFS/ME. We broadly defined CBT as a psychological intervention incorporating one or more cognitive or behavioral components, including cognitive restructuring, behavioral activation, problem‐solving, relaxation, and third‐wave components (Curtiss et al. 2021; Y. Furukawa et al. 2024; López‐López et al. 2019). Individual, group‐based, and guided self‐help delivery formats were all eligible, and we disaggregated these formats as components for the cNMA (Table 1). Control interventions included psychoeducation, relaxation, waitlist, and usual care. We defined waitlist and usual care as the minimal intervention at the treatment level. For multi‐arm trials, we included only arms that could be clearly described using the predefined components listed in Table 1. We derived the component definitions from previously published literature (Curtiss et al. 2021; T. A. Furukawa et al. 2021; Y. Furukawa et al. 2024).

**TABLE 1:** List of included components, delivery methods, and their definitions.

Components	Description
CBT component	
Cognitive restructuring (Ct)	Cognitive restructuring is a core component of cognitive behavioral therapy (CBT) that involves identifying, challenging, and modifying distorted or maladaptive thoughts and beliefs.
Behavioral activation (Ba)	Behavioral activation is a structured, evidence‐based component of CBT that aims to counter depression by increasing engagement with positively reinforcing activities, thereby reducing patterns of avoidance and withdrawal that maintain low mood.
Psychoeducation (Ps)	Education about CFS/ME symptoms and the link between behavior and mood.
Homework (Ho)	Homework refers to tasks assigned by therapists for clients to complete between sessions. These tasks are designed to help clients practice and apply the skills learned during therapy in their daily lives, facilitating the integration of new coping strategies and reinforcing behavioral changes.
Problem solving (Pr)	Problem‐solving aims to help individuals identify specific problems in their lives, generate a range of possible solutions, evaluate the pros and cons of each, and implement the most effective strategies. This process is designed to enhance coping skills, reduce distress, and promote a sense of control over challenging situations.
Social skills training (Ss)	Social skills training focuses on improving an individual's ability to interact effectively and appropriately with others.
Relaxation (Re)	The relaxation component consists of progressive muscle relaxation, basic deep breathing exercises, and general strategies for promoting physical and mental calmness.
Goal setting (Gs)	Goal setting involves collaboratively identifying specific, measurable, and achievable objectives for therapy. These goals help guide the treatment process, enhance motivation, and provide a clear sense of progress. Effective goal setting empowers clients to take an active role in their recovery and to work step‐by‐step toward meaningful change.
Third‐wave components (3w)	Third‐wave components of CBT refer to newer, mindfulness‐ and acceptance‐based approaches that extend and enrich traditional CBT. These components often emphasize changing the relationship to thoughts and emotions rather than directly modifying their content. Common third‐wave techniques include mindfulness training, acceptance strategies, cognitive defusion, values clarification, and committed action.
Delivery method	
Individual (Ind)	The CBT is provided by a therapist in a face‐to‐face individual setting.
Group (Gro)	The CBT is delivered by a therapist in a face‐to‐face group setting.
Guided self‐help (Gui)	A form of psychotherapy where a professional therapist participates in the treatment process, guiding the patient with the aid of self‐help materials that are delivered via the Internet or other media, such as books.
Others	
Waiting component (Wait)	Participants are informed that they will have the opportunity to receive an active treatment following a waiting period. If individuals assigned to the waiting list control group receive any additional components that may have therapeutic value, we will consider both the waiting period and these therapeutic elements as part of the intervention.
Nonspecific treatment effect (ns)	The impact of an intervention that arises from patients’ belief that they are receiving some form of treatment is considered a nonspecific treatment effect. In addition, various techniques that are not included in other categories and are not expected to produce significant effects are also classified as nonspecific treatment components.

We excluded trials that did not report randomization methods or trial registration, or that were quasi‐randomized. We also excluded studies that enrolled participants with idiopathic fatigue or with comorbid conditions. Trials were excluded if they investigated combination therapies (e.g., CBT combined with pharmacotherapy) or used mixed CBT delivery formats (e.g., both individual and group sessions) within the same arm.

### 2.3 Study Selection

Pairs of trained reviewers independently screened the titles, abstracts, and full texts of all identified studies. We recorded reasons for exclusion at the full‐text stage (see Appendix S2.4). Discrepancies were resolved through discussion or, if necessary, arbitration by a third reviewer.

### 2.4 Data Extraction and Risk of Bias

Pairs of reviewers extracted study characteristics, outcome data, and effect estimates for each included trial. We classified CBT treatment arms and their components according to predefined definitions (Table 1, Appendix S2.2), using all available information from the original publications. We assessed the risk of bias (ROB) using the revised Cochrane Risk of Bias tool (ROB 2.0) (Sterne et al. 2019). Disagreements were resolved through discussion or adjudicated by a third reviewer if necessary.

### 2.5 Outcomes

We extracted all patient‐important outcomes, including fatigue, anxiety, depression, physical function, sleep quality, quality of life, and pain, assessed immediately after treatment and at the end of follow‐up. For trials that reported multiple follow‐up time points, we extracted data from the longest follow‐up period.

### 2.6 Statistical Analysis

#### 2.6.1 Pairwise Meta‐Analysis

We performed random effects meta‐analysis of all direct comparisons using the DerSimonian and Laird random‐effects model. For outcomes using different measurement instruments across studies (e.g., fatigue, physical function), we harmonized treatment effects by converting scores to a common reference instrument following a previously established method (Ebrahim et al. 2014). Specifically, we converted fatigue severity to the Chalder Fatigue Questionnaire (CFQ), sleep quality to the Pittsburgh Sleep Quality Index (PSQI), physical function to the Short Form‐36 Physical Function subscale (SF‐36 PF), anxiety to the Hospital Anxiety and Depression Scale‐Anxiety subscale (HADS‐A), and depression to the HADS‐Depression subscale (HADS‐D). We calculated mean differences (MDs) with corresponding 95% confidence intervals (CIs) for continuous outcomes. Analyses were conducted separately immediately after treatment and at the end of follow‐up. When means and standard deviations (SDs) or standard errors were not reported, we estimated them from sample sizes, medians, ranges, or interquartile ranges using established methods (Luo et al. 2018; Wan et al. 2014). We assessed heterogeneity using the *I* ^2^ statistic. We evaluated potential publication bias through Egger's and Begg's tests (Begg and Mazumdar 1994; Egger et al. 1997).

#### 2.6.2 Network Meta‐Analysis

We conducted network meta‐analyses (NMA) within a frequentist framework using random‐effects models. We used the Cochran's *Q* test to evaluate the global incoherence across the network. We assessed local incoherence by comparing direct and indirect estimates within closed loops using the node‐splitting method. We further assessed incoherence between direct and indirect estimates by examining the overlap of point estimates and their corresponding 95% CIs.

#### 2.6.3 Certainty of Evidence and Interpretation of Clinical Importance

We assessed the overall certainty of evidence for network estimates using the Grading of Recommendations Assessment, Development, and Evaluation (GRADE) framework (Guyatt et al. 2008). We categorized the final certainty of evidence as high, moderate, low, or very low. For direct comparisons, the certainty of evidence started at high and could be rated down based on ROB, inconsistency, indirectness, and publication bias. For indirect comparisons, certainty was based on the lowest rating among the dominant contributing direct comparisons in the first‐order loop, with further rating down for intransitivity (Balshem et al. 2011). We assessed transitivity by comparing clinical and methodological characteristics across intervention groups; notable differences in populations or comparators led to further rating down. For network estimates, the certainty started as the certainty of the direct or indirect estimates that contributed most dominantly to the network estimate. Certainty ratings were informed by the higher rating between direct and indirect estimates when consistent, and both contributed meaningfully to the network estimate. Further rating down was applied for incoherence and imprecision. We evaluated imprecision using minimally important differences (MIDs) as thresholds specific to each outcome; detailed thresholds are listed in Figure 3. We also considered logical coherence and external indirect evidence when network estimates were based solely on indirect comparisons (Hao et al. 2023).

To facilitate clinical interpretation, we categorized treatments from least effective to most effective, considering effect size magnitude with certainty of evidence (Brignardello‐Petersen et al. 2020; Shi et al. 2023). We ranked interventions based on whether the point estimate effect and its 95% CI exceeded the MID. An intervention was considered more effective than the reference (minimal intervention) only if both the point estimate and CI surpassed the MID threshold. If the point estimate exceeded the MID but the CI included the threshold, or if the point estimate was below the MID, the intervention was categorized as not clearly superior. Based on this approach, we grouped interventions into three categories: (1) Category 1, not superior to the minimal intervention (among the worst); (2) Category 2: superior to the minimal intervention, but not superior to any other intervention in Category 2 (intermediately beneficial); and (3) Category 3: superior to at least one Category 2 intervention (among the most beneficial).

#### 2.6.4 Component Network Meta‐Analysis

We then constructed a network diagram at the component level and conducted a frequentist random‐effects cNMA. We used an additive model, assuming that the effect of a multicomponent intervention equals the sum of the individual effects of its components, with no interaction effects between components. For each component, the cNMA estimates incremental mean differences (iMDs) and corresponding 95% CIs for continuous outcomes measured using the same instruments. These estimates reflect the incremental contribution of each component to the overall effect of a multicomponent intervention. We present the effect using a color‐coded scheme to reflect the magnitude and direction based on each estimate and its 95% CI. In addition, we combined the effective components identified through cNMA and estimated the overall effect of the combined components using the additive model, with the nonspecific treatment component as the reference. The nonspecific treatment component corresponded to the usual care intervention at the treatment level, which did not include any specific cognitive or behavioral components. This comparison allowed us to assess whether the combined effect of these active components exceeded the predefined MID, indicating potential clinical benefit.

#### 2.6.5 Sensitivity and Subgroup Analyses

To assess the robustness of our findings, we conducted a series of prespecified sensitivity analyses. At the treatment level, we examined the impact of separating the minimal intervention group into waitlist and usual care. We also performed additional analyses excluding trials involving psychoeducation or relaxation. At the component level, we assessed whether the inclusion of studies with high dropout rates (≥ 20%), high overall ROB, small sample sizes (< 50 participants), or delivery format components influenced the results for fatigue. In addition, we conducted post hoc subgroup analyses to explore potential effect modifiers, including age and sex. We performed all analyses in R (version 4.3.2) and STATA (version 15.1).

## 3 Results

**FIGURE 1:**
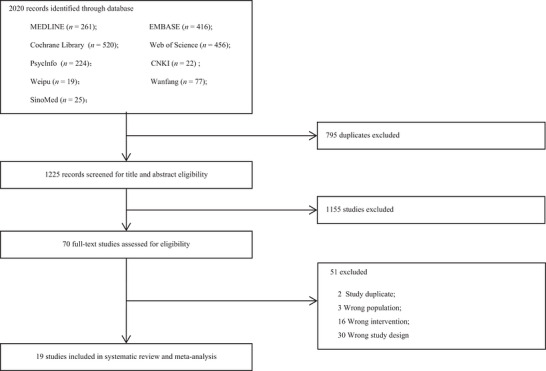
PRISMA flow diagram.

We identified a total of 2020 records through database searches. After screening titles and abstracts, we assessed 70 full‐text articles for eligibility and included 19 RCTs with 2429 participants diagnosed with CFS/ME in the systematic review (Figure 1). Of these, 16 RCTs with 2009 participants were included in the NMA. Appendix S2.1 provides a list of eligible studies.

Appendix S2.4 summarizes the characteristics of the included RCTs. The median of the reported mean ages across studies was 37.4 years (ranging from 13 to 44 years), with a median proportion of female participants of 78.2% and a median intervention duration of 24 weeks. Of the 19 included trials, 7 compared CBT with usual care, 7 with waitlist, 2 with relaxation, 2 with psychoeducation, and 1 with different CBT delivery formats. The most commonly included treatment components in the CBT arms were cognitive restructuring (20 of 21 arms [95.2%]), behavioral activation (19 of 21 arms [90.5%]), and goal setting (10 of 21 arms [47.6%]).

The overall methodological quality of the included trials was moderate, according to the ROB 2.0; 6 of 19 trials (31.6%) were rated as low ROB, 12 (63.2%) as having some concerns, and 1 (5.3%) as high risk. (Appendix S3)

### 3.1 Treatment‐Level Network Meta‐Analysis

**FIGURE 2:**
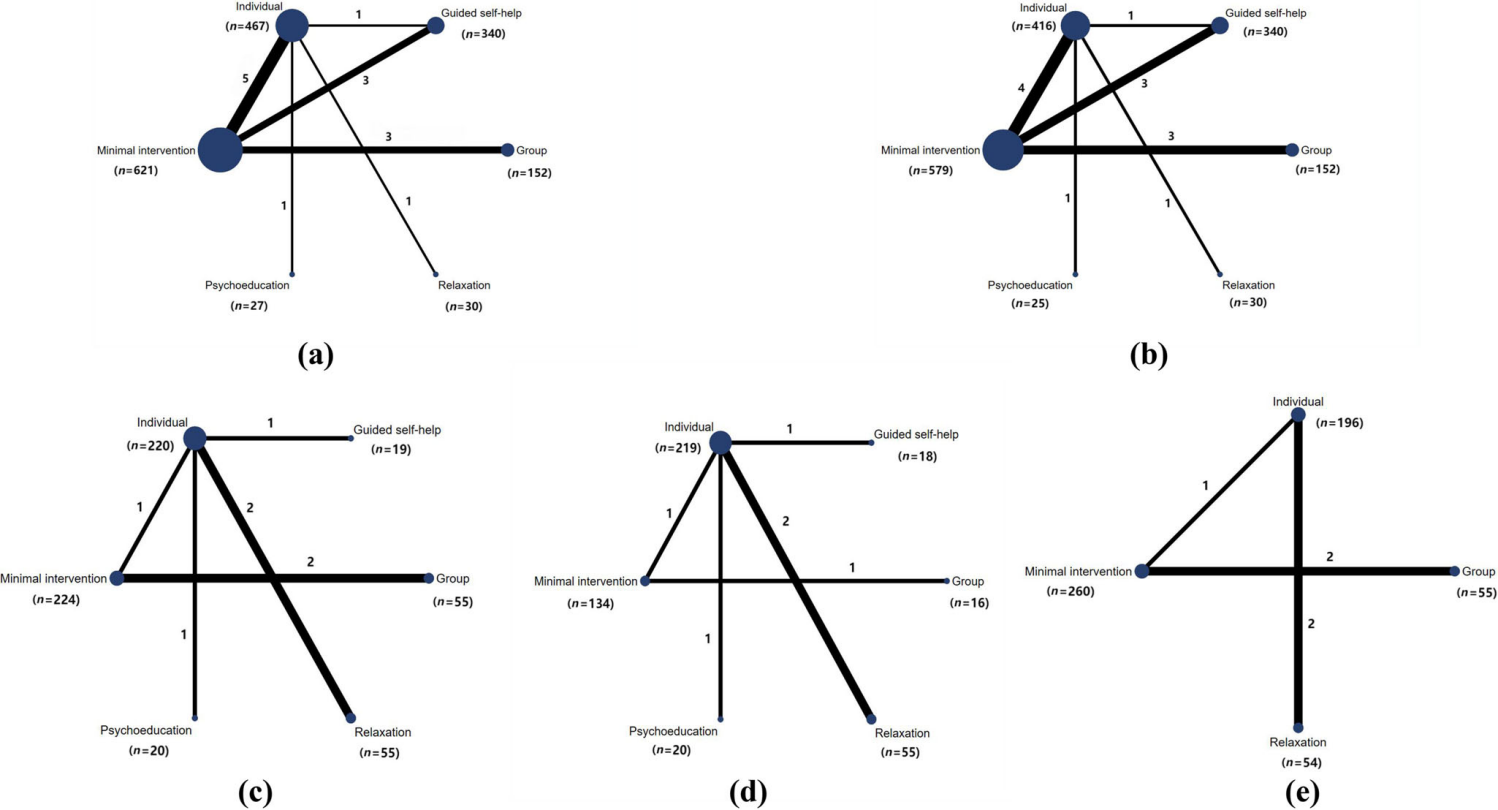
Network plots at treatment level. Network plots of eligible direct comparisons for fatigue when measured immediately after treatment (a), physical function when measured immediately after treatment (b), fatigue at the end of the follow‐up (c), physical function at the end of the follow‐up (d), and depression at the end of the follow‐up (e) outcomes. The width of the lines corresponds to the number of trials comparing each pair of interventions. The size of the nodes corresponds to the number of randomized participants.

**FIGURE 3:**
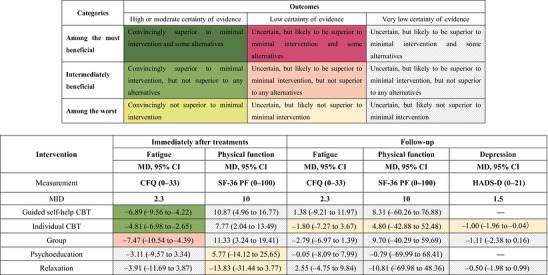
Summary of findings from the network meta‐analysis. We classified the overall certainty of evidence into four categories: “*very low*,” “*low*,” “*moderate*,” or “*high*” using the Grading of Recommendations Assessment, Development, and Evaluation (GRADE) framework. We rated down for imprecision when the 95% CI crossed MID. We rated our certainty of the benefit outcome and classified interventions into the following three categories: (1) among the most beneficial: the intervention was superior to minimal intervention and at least one active intervention (the mean effect size exceeding and the 95% CI not crossing the MID threshold); (2) intermediately beneficial: the intervention was superior to minimal intervention alone (the mean effect size exceeding and the 95% CI not crossing the MID threshold); and (3) the intervention was not superior to minimal intervention (95% CI crossing the MID threshold, or mean effect size not exceeding and the 95% CI not crossing the threshold). The categories indicate whether the effect is clinically significant, while the certainty of evidence shows how reliable the effect is. CFQ = Chalder Fatigue Scale questionnaire; CI = confidence interval; HADS‐D = Hospital Anxiety and Depression Scale‐Depression; MD = mean difference; MID = minimally important difference; SF‐36 PF = Short Form 36‐Questionnaire‐Physical Function.

Twelve trials, including 1637 participants, reported fatigue immediately after treatment. Compared with minimal intervention, guided self‐help CBT (MD, −6.89; 95% CI, −9.56 to −4.22; moderate certainty) and individual CBT (MD, −4.81; 95% CI, −6.98 to −2.65; moderate certainty) probably reduce fatigue when measured immediately after treatment. Group CBT (MD, −7.47; 95% CI, −10.54 to −4.39) may also reduce fatigue when measured immediately after treatment. Fewer studies reported on the effects of CBT at follow‐up, and we present the main results at the treatment level in Figures 2 and 3, Appendix S4.

### 3.2 Component Network Meta‐Analysis

#### 3.2.1 Effectiveness Immediately After Treatments

**FIGURE 4:**
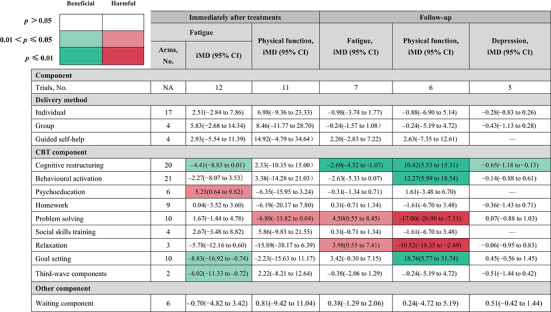
Summary of findings from the component network meta‐analysis. For fatigue, iMD < 0 denotes beneficial components (reduce fatigue) and iMD > 0 denotes harmful components (worsen fatigue). For depression, iMD < 0 denotes beneficial components (reduce depression) and iMD > 0 denotes harmful components (worsen depression). For physical function, iMD > 0 denotes beneficial components (improve physical function) and iMD < 0 denotes harmful components (reduce physical function). CI = confidence interval; iMD = incremental mean differences.

When measured immediately after treatments (median treatment time: 24 weeks), the cNMA indicated that goal setting (iMD, −8.83; 95% CI, −16.92 to −0.74), third‐wave components (iMD, −6.02; 95% CI, −11.33 to −0.72) and cognitive restructuring (iMD, −4.41; 95% CI, −8.83 to 0.01) may be beneficial in reducing fatigue, while psychoeducation (iMD, 5.23; 95% CI, 0.64 to 9.82) might be detrimental, compared with the nonspecific treatment effect component. For physical function, problem‐solving (iMD, −6.89; 95% CI, −13.82 to 0.04) was associated with a detrimental effect (Figure 4 and Appendix S5).

#### 3.2.2 Effectiveness at the End of the Follow‐Up

At the end of the follow‐up (median follow‐up: 48 weeks), cognitive restructuring emerged as the only component associated with consistent and statistically significant improvements across multiple outcomes, including fatigue (iMD, −2.69; 95% CI, −4.32 to −1.07), physical function (iMD, 10.42; 95% CI, 5.53 to 15.31), and depression (iMD, −0.65; 95% CI, −1.18 to −0.13). For physical function, goal setting (iMD, 18.76; 95% CI, 5.77 to 31.74) and behavioral activation (iMD, 12.27; 95% CI, 5.99 to 18.54) were associated with clinically meaningful benefits. However, problem‐solving (iMD, 4.50; 95% CI, 0.55 to 8.45) and relaxation (iMD, 3.98; 95% CI, 0.55 to 7.41) may be associated with increased fatigue. Problem‐solving (iMD, −17.00; 95% CI, −26.90 to −7.11) and relaxation (iMD, −10.52; 95% CI, −18.35 to −2.69) also reduced physical function (Figure 4 and Appendix S5).

Sensitivity analyses supported the robustness of the main findings. At the treatment level, findings remained consistent when the minimal intervention group was disaggregated and when trials involving psychoeducation or relaxation were excluded (see Appendix S4.6). At the component level, prespecified analyses excluding trials with high ROB or high dropout rates, small sample sizes, inactive arms, or delivery format components did not materially alter the direction or magnitude of effects across outcomes (see Appendix S5.3). Subgroup analyses did not find evidence that age or sex modified the component‐level treatment effects (see Appendix S5.4).

Under the additive cNMA framework, we estimated the expected effects of different delivery‐content combinations. Specifically, we modeled combinations of the three most beneficial treatment components—goal setting, third‐wave components, and cognitive restructuring—with each of the three CBT delivery formats. The combination delivered via guided self‐help was most effective (iMD, −16.33; 95% CI, −26.68 to −5.99) compared with the nonspecific treatment effect component, with both the point estimate and 95% CI exceeding the predefined MID threshold. Complete results of the additive model evaluating delivery‐content combinations are presented in Appendix S5.5.

## 4 Discussion

To our knowledge, this is the first component‐level NMA that systematically evaluates both delivery formats and components of CBT for CFS/ME. At the treatment level, moderate‐certainty evidence suggests that guided self‐help CBT and individual CBT probably reduce fatigue, while low‐certainty evidence indicates that group CBT may also reduce fatigue when measured immediately after treatment. At the component level, goal setting, third‐wave components, and cognitive restructuring were beneficial for fatigue, whereas psychoeducation was potentially detrimental when measured immediately after treatment. At the end of the follow‐up, cognitive restructuring was beneficial for fatigue, physical function, and depression; goal setting was associated with improved physical function. The most beneficial CBT combination for fatigue reduction may include goal setting, third‐wave components, and cognitive restructuring delivered via guided self‐help.

Our cNMA provides a clearer understanding of how individual CBT components contribute to outcomes in CFS/ME. Third‐wave components and cognitive restructuring may alleviate fatigue in patients with CFS/ME by enhancing self‐regulation, increasing perceived control, and promoting adaptive coping. Previous studies have also demonstrated that third‐wave components and cognitive restructuring are beneficial in managing chronic insomnia, suggesting a broader applicability across symptom‐based conditions (Y. Furukawa et al. 2024). In addition, goal setting, which facilitates goal‐directed behavioral activation and enhances perceived agency, may mitigate fatigue by strengthening self‐efficacy and interrupting maladaptive cycles of avoidance and deconditioning that typify the behavioral phenotype of CFS/ME.

Interestingly, improvements in physical function emerged only at the end of follow‐up for several CBT components–including cognitive restructuring, behavioral activation, and goal setting–despite no detectable effects when measured immediately after treatment. This delayed effect may reflect the enduring nature of these components, which equip patients with self‐regulatory skills that continue to be applied after therapy ends (White et al. 2011). Such sustained gains, previously observed in other trials, highlight the long‐term effects of CBT in improving functional outcomes in CFS/ME (Chalder et al. 2010; Deale et al. 1997; White et al. 2011). Furthermore, the apparent absence of immediate effects after treatment for depression may be attributable to limitations in outcome reporting, as several included trials assessed depressive symptoms only at the end of follow‐up without providing immediate posttreatment data. As a result, the lack of observed short‐term effects should be interpreted with caution, as it may reflect data sparsity rather than a true absence of therapeutic benefit.

In addition to identifying potentially beneficial components, our component‐level analysis suggested a potentially unfavorable association between psychoeducation and fatigue. One possible explanation is that psychoeducation may not function as an active therapeutic ingredient in the same way as components such as cognitive restructuring, goal setting, or third‐wave techniques. In CFS/ME, prior work suggests that reductions in focusing on fatigue (Wiborg et al. 2011) and fear‐avoidance beliefs (Chalder et al. 2015), as well as changes in some avoidance‐related beliefs or behaviors (Chalder et al. 2015; Deale et al. 1998), are associated with better outcomes after CBT or related rehabilitative interventions. In addition, illness‐specific attentional and interpretive biases may contribute to symptom persistence by reinforcing unhelpful illness beliefs and behaviors (Hughes et al. 2017). Accordingly, when psychoeducation is delivered without sufficient cognitive or behavioral change strategies, it may be less likely to modify these maintaining factors and may, in some contexts, inadvertently increase symptom monitoring. However, this finding should be interpreted cautiously. The observed signal may reflect how psychoeducation was framed, delivered, or embedded within particular treatment packages, rather than implying that psychoeducation is inherently harmful.

While delivery formats such as guided self‐help and individual CBT were associated with improvements in fatigue at the treatment level, consistent with prior meta‐analyses, our cNMA did not identify any delivery format components that were consistently effective across outcomes (Kuut et al. 2024b). This discrepancy suggests that the effectiveness of delivery formats may depend more on the therapeutic content they convey than on the mode of delivery itself. When separated from specific intervention elements, delivery formats may exert a limited independent influence on treatment outcomes. Notably, CBT incorporating goal setting, third‐wave components, and cognitive restructuring delivered in a guided self‐help format was associated with significant improvement in fatigue, highlighting that delivery formats may augment treatment effects when aligned with specific effective components, rather than acting as independent mechanisms.

### 4.1 Strengths and Limitations

This study has several key strengths. To our knowledge, it is the first cNMA to systematically evaluate both delivery formats and treatment components of CBT for CFS/ME. By integrating traditional and component‐level NMA, we provided a comprehensive assessment of CBT effectiveness. The analysis identified not only effective formats and components, but also a promising combination for reducing fatigue (goal setting, third‐wave components, and cognitive restructuring delivered via guided self‐help), as well as potentially detrimental components (psychoeducation and problem‐solving). These findings may guide more targeted CBT strategies in CFS/ME management. Identification of delivery and content components played a central role in our analysis. All components were carefully defined based on intervention descriptions and systematically coded across studies. We used the GRADE approach to assess the certainty of evidence at the NMA level, which is rarely done in previously published cNMA studies. In addition, we applied MID thresholds to assess whether the estimated effects, including those of combined components, were clinically meaningful.

However, several limitations should be noted. First, several secondary outcomes, particularly depression when measured immediately after treatment, were inadequately reported. This was partly due to some trials only reporting follow‐up outcomes, which may have reduced the precision of effect estimates and limited temporal comparability. Second, the analysis assumed additive effects across CBT components. Although previous cNMAs have not identified strong evidence against additivity (T. A. Furukawa et al. 2021; Y. Furukawa et al. 2024; López‐López et al. 2019; Pompoli et al. 2018), this assumption may not fully reflect real‐world interactions, where therapeutic components can be synergistic or antagonistic. In addition, because intervention components were not randomly distributed across studies, residual confounding by study‐level contextual factors cannot be excluded. While we attempted to explore and mitigate this risk through transitivity assessments, sensitivity analyses, and subgroup analyses for demographic variables (age and sex), which did not materially alter our main findings, the potential for residual confounding remains. Accordingly, the component‐level estimates should be interpreted cautiously as component‐associated effects within the cNMA framework rather than as definitive causal effects of individual components. Third, we coded CBT components dichotomously as present or absent based on how they were described in the included studies. In practice, the content and degree of implementation of components may have differed across programs. Fourth, because multiple component‐level estimates were generated within the cNMA, *p*‐values should be interpreted cautiously to avoid the risk of spurious findings from multiplicity. In our interpretation, we prioritized the magnitude of effect estimates, their precision (95% CIs), and their consistency, rather than relying solely on statistical significance. Accordingly, the present analyses should be viewed as exploratory and hypothesis‐generating rather than confirmatory or conclusive. Larger and methodologically rigorous trials are needed to determine whether specific component combinations yield superior outcomes. Our cNMA highlights components that may warrant further investigation.

## 5 Conclusion

The findings of this study suggest that the most beneficial CBT combination may include goal setting, third‐wave components, and cognitive restructuring delivered via guided self‐help. Behavioral activation may be nonessential, while psychoeducation may be detrimental. Future studies should prospectively examine core CBT components, assess their interactions, and incorporate measures of intensity to guide the development of more personalized and scalable interventions.

## Author Contributions

**Qiukui Hao**: methodology, validation, investigation, formal analysis, writing – review and editing. **Yu‐Qing Zhang**: methodology, formal analysis, writing – review and editing. **Shoujian Wang**: methodology, writing – review and editing, writing – original draft, formal analysis, validation. **Jun Ren**: methodology, validation, visualization, writing – review and editing, formal analysis, writing – original draft. **Min Fang**: writing – review and editing, investigation, conceptualization, project administration, validation, supervision. **Sitong Fang**: methodology, validation, investigation, formal analysis. **Lingjun Kong**: writing – review and editing, supervision, validation, conceptualization, investigation, funding acquisition.

## Funding

This work was supported by the Shanghai Key Laboratory of Traditional Chinese Medicine Manipulation Therapy for Musculoskeletal Diseases (24dz2260200), Shuguang Hospital Affiliated to Shanghai University of Traditional Chinese Medicine—Research Leadership Initiative (SGYYJBGS‐001), the Shanghai Three‐Year Action Plan for Advancing TCM Inheritance and Innovation (ZY(2025‐2027)‐3‐1‐1), the Shanghai Central‐Local Joint Science and Technology Development Fund (YDZX20243100002004), and the Shanghai Pudong New Area Health Commission Special Project (PW2023E‐01).

## Ethics Statement

The authors have nothing to report.

## Conflicts of Interest

The authors declare no conflicts of interest.

## Supporting information




**Supplementary materials**: brb371513‐sup‐0001‐AppendixS1‐S5.pdf

## Data Availability

Data that support the findings of this study are available from the corresponding author, Min Fang, upon reasonable request.
