# Supplementary material for: Components and Delivery Formats of Cognitive Behavioral Therapy for Chronic Fatigue Syndrome/Myalgic Encephalomyelitis: A Systematic Review and Component Network Meta‐Analysis
Source: Brain Behav. 2026 May 31;16(6):e71513. doi: 10.1002/brb3.71513 (PMC13239200; doi:10.1002/brb3.71513)
Supplement: Supplementary file 1 — Supplementary materials: brb371513‐sup‐0001‐AppendixS1‐S5.pdf [file BRB3-16-e71513-s001.pdf]

Supplementary material

Appendix S1: Search strategy 1
S1.1 MEDLINE (OVID) 1
S1.2 EMBASE (OVID) 3
S1.3 PsycInfo (OVID) 4
S1.4 Cochrane Library 5
S1.5 Web of Science 6
S1.6 China Knowledge Resource Integrated Database 7
S1.7 Wanfang Data Information 7
S1.8 Weipu Database for Chinese Technical Periodicals 8
S1.9 Chinese BioMedical Literature databases (SinoMed) 8
Appendix S2: Summary of included studies 9
S2.1 Reference list of included studies 9
S2.2 Definitions of Included Interventions 11
S2.3 Conceptualization of CBT for CFS/ME or control conditions from the component perspective 12
S2.4 Characteristics of included studies 13
S2.5 Summary of excluded studies 17
Appendix S3: Risk of bias assessments 23
Appendix S4 Main results at treatment level 28
Appendix S4.1 Network estimates (league tables) 28
S4.1.1 Fatigue when measured immediately after treatments 29
S4.1.2 Physical function when measured immediately after treatments 30
S4.1.3 Fatigue at the end of the follow-up 31
S4.1.4 Physical function at the end of the follow-up 32

S4.1.5 Depression at the end of the follow-up 33
Appendix S4.2 Forest plots 34
S4.2.1 Fatigue when measured immediately after treatments 34
S4.2.2 Physical function when measured immediately after treatments 35
S4.2.3 Fatigue at the end of the follow-up 36
S4.2.4 Physical function at the end of the follow-up 37
S4.2.5 Depression at the end of the follow-up 38
Appendix S4.3 Heterogeneity 39
S4.3.1 Fatigue when measured immediately after treatments 39
S4.3.2 Physical function when measured immediately after treatments 40
S4.3.3 Fatigue at the end of the follow-up 41
S4.3.4 Physical fnction at the end of the follow-up 42
S4.3.5 Depression at the end of the follow-up 43
Appendix S4.4 Publication bias (Begger or Egger's test) 44
S4.4.1 Fatigue when measured immediately after treatments 44
S4.4.2 Physical function when measured immediately after treatments 44
S4.4.3 Fatigue at the end of the follow-up 45
S4.4.4 Physical function at the end of the follow-up 45
S4.4.5 Depression at the end of the follow-up 45
Appendix S4.5: GRADE assessments: certainty of evidence for direct, indirect and network estimates 46
S4.5.1 Fatigue when measured immediately after treatments 46
S4.5.2 Physical function when measured immediately after treatments 49
S4.5.3 Fatigue at the end of the follow-up 52
S4.5.4 Physical function at the end of the follow-up 55
S4.5.5 Depression at the end of the follow-up 58
Appendix S4.6:Sensitivity analyses 60

S4.6.1 Sensitivity analyses by separating the minimal intervention group into waitlist and usual care. 60
S4.6.2 Sensitivity analyses by excluding trials involving psychoeducation or relaxation. 61
Appendix S5: Main results at component level 62
S5.1 Network plots 63
5.1.1 Fatigue when measured immediately after treatments 63
5.1.2 Physical function when measured immediately after treatments 64
5.1.3 Fatigue at the end of the follow-up 65
5.1.4 Physical function at the end of the follow-up 66
5.1.5 Depression at the end of the follow-up 67
S5.2 Forest plots 68
5.2.1 Fatigue when measured immediately after treatments 68
5.2.2 Physical function when measured immediately after treatments 69
5.2.3 Fatigue at the end of the follow-up 70
5.2.4 Physical function at the end of the follow-up 71
5.2.5 Depression at the end of the follow-up 72
S5.3 Sensitivity analyses 73
S5.4 Subgroup analyses 74
S5.5 The additive model evaluating delivery–content combinations 75

Appendix S1: Search strategy

S1.1 MEDLINE (OVID)

1. Fatigue Syndrome, Chronic/
2. (chronic adj3 fatigue).mp. [mp=title, book title, abstract, original title, name of substance word, subject heading word, floating sub-heading word, keyword heading word, organism supplementary concept word, protocol supplementary concept word, rare disease supplementary concept word, unique identifier, synonyms]
3. (fatigue adj3 syndrome).mp. [mp=title, book title, abstract, original title, name of substance word, subject heading word, floating sub-heading word, keyword heading word, organism supplementary concept word, protocol supplementary concept word, rare disease supplementary concept word, unique identifier, synonyms]
4. (myalgic adj3 encephalomyelitis).mp. [mp=title, book title, abstract, original title, name of substance word, subject heading word, floating sub-heading word, keyword heading word, organism supplementary concept word, protocol supplementary concept word, rare disease supplementary concept word, unique identifier, synonyms]
5. CFS.mp.
6. Royal free disease.mp.
7. fatigue- fatigue-fibromyalgia.mp.
8. (chronic adj3 mononucleosis).mp. [mp=title, book title, abstract, original title, name of substance word, subject heading word, floating sub-heading word, keyword heading word, organism supplementary concept word, protocol supplementary concept word, rare disease supplementary concept word, unique identifier, synonyms]
9. (chronic adj3 Epstein Barr).mp. [mp=title, book title, abstract, original title, name of substance word, subject heading word, floating sub-heading word, keyword heading word, organism supplementary concept word, protocol supplementary concept word, rare disease supplementary concept word, unique identifier, synonyms]
10. systemic exertion intolerance.mp.
11. epidemic neuromyasthenia.mp.
12. CFIDS.mp.
13. PVFS.mp.
14. Yuppie.mp.
15. iceland disease.mp.
16. akureyri disease.mp.
17. or/1-16
18. Cognitive Behavioral Therapy.mp. or exp Cognitive Behavioral Therapy/
19. Behavioral Therapies, Cognitive OR Behavioral Therapy, Cognitive OR Cognitive Behavioral Therapies OR Therapies, Cognitive Behavioral OR Therapy, Cognitive Behavioral OR Behavior Therapy, Cognitive OR Cognitive Behavior Therapy OR Cognitive Behaviour Therapy OR Behaviour Therapies, Cognitive OR Behaviour Therapy, Cognitive OR Cognitive Behaviour Therapies OR Therapies, Cognitive Behaviour OR Therapy, Cognitive Behaviour OR Cognitive Therapy OR Therapy, Cognitive Behavior OR Behavior Therapies, Cognitive OR Cognitive Behavior Therapies OR Therapies, Cognitive Behavior OR Cognitive Psychotherapy OR Cognitive

Psychotherapies OR Psychotherapies, Cognitive OR Psychotherapy, Cognitive OR Therapy, Cognitive OR Cognitive Therapies OR Therapies, Cognitive OR Therapy, Cognition OR Cognition Therapy OR Cognition Therapies OR Therapies, Cognition
20. 18 or 19
21. 17 and 20
22. randomized controlled trial.pt.
23. controlled clinical trial.pt.
24. randomi?ed.ab.
25. placebo.ab.
26. randomly.ab.
27. trial.ab.
28. groups.ab.
29. or/22-28
30. 21 and 29
31. exp animals/ not humans.sh.
32. 30 not 31

S1.2 EMBASE (OVID)

1.Fatigue Syndrome, Chronic/
2. (chronic adj3 fatigue).mp. [mp=title, book title, abstract, original title, name of substance word, subject heading word, floating sub-heading word, keyword heading word, organism supplementary concept word, protocol supplementary concept word, rare disease supplementary concept word, unique identifier, synonyms]
3. (fatigue adj3 syndrome).mp. [mp=title, book title, abstract, original title, name of substance word, subject heading word, floating sub-heading word, keyword heading word, organism supplementary concept word, protocol supplementary concept word, rare disease supplementary concept word, unique identifier, synonyms]
4. (myalgic adj3 encephalomyelitis).mp. [mp=title, book title, abstract, original title, name of substance word, subject heading word, floating sub-heading word, keyword heading word, organism supplementary concept word, protocol supplementary concept word, rare disease supplementary concept word, unique identifier, synonyms]
5. CFS.mp.
6. Royal free disease.mp.
7. fatigue- fatigue-fibromyalgia.mp.
8. (chronic adj3 mononucleosis).mp. [mp=title, book title, abstract, original title, name of substance word, subject heading word, floating sub-heading word, keyword heading word, organism supplementary concept word, protocol supplementary concept word, rare disease supplementary concept word, unique identifier, synonyms]
9. (chronic adj3 Epstein Barr).mp. [mp=title, book title, abstract, original title, name of substance word, subject heading word, floating sub-heading word, keyword heading word, organism supplementary concept word, protocol supplementary concept word, rare disease supplementary concept word, unique identifier, synonyms]
10. systemic exertion intolerance.mp.
11. epidemic neuromyasthenia.mp.
12. CFIDS.mp.
13. PVFS.mp.
14. Yuppie.mp.
15. iceland disease.mp.
16. akureyri disease.mp.
17. or/1-16
18. exp behavior therapy/ or exp cognitive behavioral therapy/ or exp cognitive therapy/
19. Behavioral Therapies, Cognitive OR Behavioral Therapy, Cognitive OR Cognitive Behavioral Therapies OR Therapies, Cognitive Behavioral OR Therapy, Cognitive Behavioral OR Behavior Therapy, Cognitive OR Cognitive Behavior Therapy OR Cognitive Behaviour Therapy OR Behaviour Therapies, Cognitive OR Behaviour Therapy, Cognitive OR Cognitive Behaviour Therapies OR Therapies, Cognitive Behaviour OR Therapy, Cognitive Behaviour OR Cognitive Therapy OR Therapy, Cognitive Behavior OR Behavior Therapies, Cognitive OR Cognitive Behavior Therapies OR Therapies, Cognitive Behavior OR Cognitive Psychotherapy OR Cognitive Psychotherapies OR Psychotherapies, Cognitive OR Psychotherapy, Cognitive OR Therapy, Cognitive OR Cognitive Therapies OR Therapies, Cognitive OR Therapy, Cognition OR Cognition Therapy OR Cognition Therapies OR Therapies, Cognition
20. 18 or 19
21. 17 and 20

22. randomized controlled trial.pt.
23. controlled clinical trial.pt.
24. randomi?ed.ab.
25. placebo.ab.
26. randomly.ab.
27. trial.ab.
28. groups.ab.
29. or/22-28
30. 21 and 29
31. animals.mp. or animal/
32. 30 not 31

S1.3 PsycInfo (OVID)

1. chronic fatigue syndrome/
2. (chronic adj3 fatigue).mp. [mp=title, abstract, heading word, table of contents, key concepts, original title, tests & measures, mesh word]
3. (fatigue adj3 syndrome).mp. [mp=title, abstract, heading word, table of contents, key concepts, original title, tests & measures, mesh word]
4. (myalgic adj3 encephalomyelitis).mp. [mp=title, abstract, heading word, table of contents, key concepts, original title, tests & measures, mesh word]
5. CFS.mp.
6. Royal free disease.mp.
7. fatigue-fibromyalgia.mp.
8. (chronic adj3 mononucleosis).mp. [mp=title, abstract, heading word, table of contents, key concepts, original title, tests & measures, mesh word]
9. (chronic adj3 Epstein Barr).mp. [mp=title, abstract, heading word, table of contents, key concepts, original title, tests & measures, mesh word]
10. systemic exertion intolerance.mp.
11. epidemic neuromyasthenia.mp.
12. CFIDS.mp.
13. PVFS.mp.
14. Yuppie.mp.
15. iceland disease.mp.
16. akureyri disease.mp.
17. or/1-16
18. exp Cognitive Behavior Therapy/ or exp Cognitive Therapy/ or exp Behavior Therapy/ or Cognitive Behavioral Therapy.mp.
19. Behavioral Therapies, Cognitive OR Behavioral Therapy, Cognitive OR Cognitive Behavioral Therapies OR Therapies, Cognitive Behavioral OR Therapy, Cognitive Behavioral OR Behavior Therapy, Cognitive OR Cognitive Behavior Therapy OR Cognitive Behaviour Therapy OR Behaviour Therapies, Cognitive OR Behaviour Therapy,

Cognitive OR Cognitive Behaviour Therapies OR Therapies, Cognitive Behaviour OR Therapy, Cognitive Behaviour OR Cognitive Therapy OR Therapy, Cognitive Behavior OR Behavior Therapies, Cognitive OR Cognitive Behavior Therapies OR Therapies, Cognitive Behavior OR Cognitive Psychotherapy OR Cognitive Psychotherapies OR Psychotherapies, Cognitive OR Psychotherapy, Cognitive OR Therapy, Cognitive OR Cognitive Therapies OR Therapies, Cognitive OR Therapy, Cognition OR Cognition Therapy OR Cognition Therapies OR Therapies, Cognition

20. 18 or 19
21. 17 and 20
22. (double-blind or random: assigned or control).tw.
23. clinical trials/
24. (controlled adj3 trial*).mp. [mp=title, abstract, heading word, table of contents, key concepts, original title, tests & measures, mesh word]
25. (clinical adj2 trial*).mp. [mp=title, abstract, heading word, table of contents, key concepts, original title, tests & measures, mesh word]
26. (randomi?ed adj7 trial*).mp. [mp=title, abstract, heading word, table of contents, key concepts, original title, tests & measures, mesh word]
27. or/22-26
28. 21 and 27

S1.4 Cochrane Library

1. MeSH descriptor: [Fatigue Syndrome, Chronic] explode all trees
2. chronic near/3 fatigue
3. fatigue near/3 syndrome
4. myalgic near/3 encephalomyelitis
5. CFS
6. Royal free disease
7. fatigue-fibromyalgia
8. chronic near/3 mononucleosis
9. chronic near/3 Epstein Barr
10. systemic exertion intolerance
11. epidemic neuromyasthenia
12. CFIDS
13. PVFS
14. Yuppie
15. iceland disease.mp.
16. akureyri disease.mp.
17. #1 or #2 or #3 or #4 or #5 or #6 or #7 or #8 or #9 or #10 or #11 or #12 or #13 or #14 or #15 or #16

18. Cognitive Behavioral Therapy
19. Cognition Therapies or Cognitive Behavior Therapies or Therapy, Cognitive Behavior or Behavior Therapy, Cognitive or Cognitive Psychotherapy or Behaviour Therapy, Cognitive or Psychotherapies, Cognitive or Therapies, Cognitive Behavioral or Therapy, Cognition or Cognitive Behavior Therapy or Cognitive Behaviour Therapies or Therapies, Cognitive or Cognition Therapy or Therapies, Cognition or Behaviour Therapies, Cognitive or Behavioral Therapies, Cognitive or Cognitive Therapy or Cognitive Behaviour Therapy or Therapy, Cognitive Behavioral or Therapy, Cognitive or Therapies, Cognitive Behavior or Psychotherapy, Cognitive or Therapy, Cognitive Behaviour or Cognitive Therapies or Behavior Therapies, Cognitive or Cognitive Behavioral Therapies or Behavioral Therapy, Cognitive or Therapies, Cognitive Behaviour or Cognitive Psychotherapies
20. #18 or #19
21. #17 and #20
22. randomized controlled trial
23. Trials, Randomized Clinical or Clinical Trials, Randomized or Controlled Clinical Trials, Randomized
24. #22 or #23
25 #21 and #24 in Trials

S1.5 Web of Science

25. #21 and #24
24. #22 or #23
23. TS=(randomized controlled study OR randomized controlled trial OR randomized study OR
randomized trial OR randomized placebo-controlled study OR randomized placebo
controlled trial OR randomized placebo controlled OR randomized placebo-controlled OR
randomized double-blin* OR randomized double blin* OR randomized AND double-blin*
OR randomized AND placebo-controlled)
22. TS=(Randomized Controlled Trials)
21. #17 and #20
20. #18 or #19
19. TS=(Behavioral Therapies, Cognitive OR Behavioral Therapy, Cognitive OR Cognitive Behavioral Therapies OR Therapies, Cognitive Behavioral OR Therapy, Cognitive Behavioral OR Behavior Therapy, Cognitive OR Cognitive Behavior Therapy OR Cognitive Behaviour Therapy OR Behaviour Therapies, Cognitive OR Behaviour Therapy, Cognitive OR Cognitive Behaviour Therapies OR Therapies, Cognitive Behaviour OR Therapy, Cognitive Behaviour OR Cognitive Therapy OR Therapy, Cognitive Behavior OR Behavior Therapies, Cognitive OR Cognitive Behavior Therapies OR Therapies, Cognitive Behavior OR Cognitive Psychotherapy OR Cognitive Psychotherapies OR Psychotherapies, Cognitive OR Psychotherapy, Cognitive OR Therapy, Cognitive OR Cognitive Therapies OR Therapies, Cognitive OR Therapy, Cognition OR Cognition Therapy OR Cognition Therapies OR Therapies, Cognition)
18. TS=(Cognitive Behavioral Therapy)

17. #1 or #2 or #3 or #4 or #5 or #6 or #7 or #8 or #9 or #10 or #11 or #12 or #13 or #14 or #15 or #16
16. TS=(akureyri disease)
15. TS=(iceland disease)
14. TS=(Yuppie)
13. TS=(PVFS)
12. TS=(CFIDS)
11. TS=(royal free disease)
10. TS=(epidemic neuromyasthenia)
9. TS=(systemic exertion intolerance)
8. TS=(chronic Near/3 Epstein Barr)
7. TS=(chronic NEAR/3 mononucleosis)
6. TS=(fatigue-fibromyalgia)
5. TS=(CFS)
4. TS=(myalgic near/3 encephalomyelitis)
3. TS=(fatigue near/3 syndrome)
2. TS=(chronic Near/3 fatigue)
1. TS=(chronic fatigue syndrome)

S1.6 China Knowledge Resource Integrated Database

#1 (主题=慢性疲劳综合征) OR (主题=肌痛性脑脊髓炎) OR (主题=慢性疲劳) OR (主题=慢性疲劳和免疫功能障碍综合征)
#2 (主题=认知行为疗法) OR (主题=认知行为干预) OR (主题=认知行为治疗) OR (主题=认知疗法) OR (主题=行为疗法) OR (主题=认知治疗) OR (主题=认知心理疗法) OR (主题=CBT)
#3 #1 AND #2

S1.7 Wanfang Data Information

主题=(慢性疲劳综合征 OR 肌痛性脑脊髓炎 OR 慢性疲劳 OR 慢性疲劳和免疫功能障碍综合征) and 主题=(认知行为疗法 OR 认知行为干预 OR 认知行为治疗 OR 认知疗法 OR 行为疗法 OR 认知治疗 OR 认知心理疗法 OR CBT) and 主题=(随机 OR 随机对照 OR RCT)

S1.8 Weipu Database for Chinese Technical Periodicals

主题=(慢性疲劳综合征 OR 肌痛性脑脊髓炎 OR 慢性疲劳 OR 慢性疲劳和免疫功能障碍综合征) and 主题=(认知行为疗法 OR 认知行为干预 OR 认知行为治疗 OR 认知疗法 OR 行为疗法 OR 认知治疗 OR 认知心理疗法 OR CBT)

S1.9 Chinese BioMedical Literature databases (SinoMed)

("慢性疲劳综合征"[摘要:智能] OR "肌痛性脑脊髓炎"[摘要:智能] OR "慢性疲劳"[摘要:智能] OR "慢性疲劳和免疫功能障碍综合征"[摘要:智能]) AND("认知行为疗法"[摘要:智能] OR "认知行为干预"[摘要:智能] OR "认知行为治疗"[摘要:智能] OR "认知疗法"[摘要:智能] OR "行为疗法"[摘要:智能] OR "认知治疗"[摘要:智能] OR "认知心理疗法"[摘要:智能] OR "CBT"[摘要:智能] AND ("随机对照试验"[文献类型] AND "人类"[特征词])

Appendix S2: Summary of included studies

S2.1 Reference list of included studies

1. Burgess M, Andiappan M, Chalder T. Cognitive behaviour therapy for chronic fatigue syndrome in adults: face to face versus telephone treatment: a randomized controlled trial. Behav Cogn Psychother. 2012;40(2):175-191. doi:10.1017/S1352465811000543

2. Chalder T, Deary V, Husain K, Walwyn R. Family-focused cognitive behaviour therapy versus psycho-education for chronic fatigue syndrome in 11- to 18-year-olds: a randomized controlled treatment trial. Psychol Med. 2010;40(8):1269-1279. doi:10.1017/S003329170999153X

3. Deale A, Chalder T, Marks I, Wessely S. Cognitive behavior therapy for chronic fatigue syndrome: a randomized controlled trial. Am J Psychiatry. 1997;154(3):408-414. doi:10.1176/ajp.154.3.408

4. Gotaas ME, Stiles TC, Bjørngaard JH, Borchgrevink PC, Fors EA. Cognitive Behavioral Therapy Improves Physical Function and Fatigue in Mild and Moderate Chronic Fatigue Syndrome: A Consecutive Randomized Controlled Trial of Standard and Short Interventions . Front Psychiatry. 2021;12:580924. Published 2021 Apr 12 doi:10.3389/fpsyt.2021.580924.

5. Janse A, Worm-Smeitink M, Bleijenberg G, Donders R, Knoop H. Efficacy of web-based cognitive-behavioural therapy for chronic fatigue syndrome: randomised controlled trial. Br J Psychiatry. 2018;212(2):112-118. doi:10.1192/bjp.2017.22

6. O'Dowd H, Gladwell P, Rogers CA, Hollinghurst S, Gregory A. Cognitive behavioural therapy in chronic fatigue syndrome: a randomised controlled trial of an outpatient group programme. Health Technol Assess. 2006;10(37):iii-121. doi:10.3310/hta10370

7. Knoop H, van der Meer JW, Bleijenberg G. Guided self-instructions for people with chronic fatigue syndrome: randomised controlled trial. Br J Psychiatry. 2008;193(4):340-341. doi:10.1192/bjp.bp.108.051292

8. Lloyd AR, Hickie I, Brockman A, et al. Immunologic and psychologic therapy for patients with chronic fatigue syndrome: a double-blind, placebo-controlled trial. Am J Med. 1993;94(2):197-203. doi:10.1016/0002-9343(93)90183-p

9. Lloyd S, Chalder T, Rimes KA. Family-focused cognitive behaviour therapy versus psycho-education for adolescents with chronic fatigue syndrome: long-term follow-up of an RCT. Behav Res Ther. 2012;50(11):719-725. doi:10.1016/j.brat.2012.08.005

10. Nijhof SL, Bleijenberg G, Uiterwaal CS, Kimpen JL, van de Putte EM. Effectiveness of internet-based cognitive behavioural treatment for adolescents with chronic fatigue syndrome (FITNET): a randomised controlled trial. Lancet. 2012;379(9824):1412-1418. doi:10.1016/S0140-6736(12)60025-7

11. Prins JB, Bleijenberg G, Bazelmans E, et al. Cognitive behaviour therapy for chronic fatigue syndrome: a multicentre randomised controlled trial. Lancet. 2001;357(9259):841-847. doi:10.1016/S0140-6736(00)04198-2
12. Rimes KA, Wingrove J. Mindfulness-based cognitive therapy for people with chronic fatigue syndrome still experiencing excessive fatigue after cognitive behaviour therapy: a pilot randomized study. Clin Psychol Psychother. 2013;20(2):107-117. doi:10.1002/cpp.793
13. Sharpe M, Goldsmith KA, Johnson AL, Chalder T, Walker J, White PD. Rehabilitative treatments for chronic fatigue syndrome: long-term follow-up from the PACE trial. Lancet Psychiatry. 2015;2(12):1067-1074. doi:10.1016/S2215-0366(15)00317-X
14. Sharpe M, Hawton K, Simkin S, et al. Cognitive behaviour therapy for the chronic fatigue syndrome: a randomized controlled trial. BMJ. 1996;312(7022):22-26. doi:10.1136/bmj.312.7022.22
15. Stulemeijer M, de Jong LW, Fiselier TJ, Hoogveld SW, Bleijenberg G. Cognitive behaviour therapy for adolescents with chronic fatigue syndrome: randomised controlled trial [published correction appears in BMJ. 2005 Apr 9;330(7495):820]. BMJ. 2005;330(7481):14. doi:10.1136/bmj.38301.587106.63
16. White PD, Goldsmith KA, Johnson AL, et al. Comparison of adaptive pacing therapy, cognitive behaviour therapy, graded exercise therapy, and specialist medical care for chronic fatigue syndrome (PACE): a randomised trial. Lancet. 2011;377(9768):823-836. doi:10.1016/S0140-6736(11)60096-2
17. Wiborg JF, van Bussel J, van Dijk A, Bleijenberg G, Knoop H. Randomised controlled trial of cognitive behaviour therapy delivered in groups of patients with chronic fatigue syndrome. Psychother Psychosom. 2015;84(6):368-376. doi:10.1159/000438867
18. Al-Haggar M, Al-Naggar ZA, Abdel-Salam MA. Biofeedback and cognitive behavioral therapy for Egyptian adolescents suffering from chronic fatigue syndrome. J Pediatr Neurol.2006;4:161-169.
19. Jason LA, Torres-Harding S, Friedberg F, et al. Non-pharmacologic Interventions for CFS: A Randomized Trial. J Clin Psychol Med Settings. 2007;14(4):275-296. doi:10.1007/s10880-007-9090-7

S2.2 Definitions of Included Interventions

**Intervention categories**	**Definition**
Individual CBT	The CBT is provided by the therapist in a face-to-face individual setting.
Group CBT	The CBT is delivered by the therapist in a face-to-face group setting.
Guided self-help CBT	A form of psychotherapy where a professional therapist participates in the treatment process, guiding the patient with the aid of self-help materials that are delivered via the internet or other media, such as books.
Psychoeducation	Psychoeducation is a structured approach that provides individuals with information and psychological support to help them understand and manage their condition.
Relaxation	Relaxation includes progressive muscle relaxation, basic deep breathing techniques, and general relaxation strategies.
Minimal intervention	Including waitlist, usual care, specialist medical care, routine care, and conventional pharmacological treatment.

S2.3 Conceptualization of CBT for CFS/ME or control conditions from the component perspective

**Intervention categories**	**Possible combinations of components**
Individual CBT	+ Ind ± Ct ± Ba ± Ps ± Ho ± Pr ± Ss ± Re ± Gs ± 3w
Group CBT	+ Gro ± Ct ± Ba ± Ps ± Ho ± Pr ± Ss ± Re ± Gs ± 3w
Guided self-help CBT	+ Gui ± Ct ± Ba ± Ps ± Ho ± Pr ± Ss ± Re ± Gs ± 3w
Psychoeducation	+ Ps ± Ind ± Gro ± Dig ± Gui ± Ung ± Ho ± Ss ± Re ± Gs
Relaxation	+ Re ± Ind ± Gro ± Dig ± Gui ± Ung ± Pr ± Ss ± Gs ± Ho
Usual care	+ Ns
Waitlist	+ Wait

Components marked with a " + " are required. Components marked with " ± " are optional. Components not mentioned cannot be included.

Abbreviations: Ind=Individual. Gro=Group. Gui=Guided self-help. Ct=Cognitive restructuring. Ba=Behavioural activation. Ps=Psychoeducation. Ho=Homework. Pr=Problem solving. Ss=Social skills training. Re=Relaxation. Gs=Goal setting. 3w=Third-wave components

S2.4 Characteristics of included studies

Study ID	Country	Diagnostic criteria	Intervention	Components of CBT	Components of the control group	No. of patients	Mean age (years)	Female (%)	Duration of illness (weeks)	Duration of intervention (weeks)	Follow-up (weeks)	Outcomes
Burgess 2012	England	CDC /Oxford	Guided self-help CBT VS Individual CBT	Gui+Ct+Ba+Ho +Gs	Ind+Ct+Ba+Ho+ Gs	80	37.4	78.8	NR	26	48	Fatigue, physical function
Sharpe 2015	UK	Oxford criteria	Individual CBT VS Usual care	Ind+Ct+Ba+Pr	ns	321	38.0	78.2	129	24	31 months (IQR 30–32; range 24–53).	Fatigue, physical function
Deale 1997	UK	Oxford and CDC criteria	Individual CBT VS Relaxation	Ind+Ct+Ba+Ho	Ind+Ho+Re	60	35.0	68.3	192	16-24	24	Fatigue, physical function, quality of life, depression, anxiety
Stulemeijer 2005	Netherlands	CDC criteria	Individual CBT VS Waitlist	Ind+Ct+Ba+3w	Wait	69	16.0	89.9	68	20	NR	Fatigue, physical

												function
Nijhof 2012	Netherlands	CDC criteria	Guided self-help CBT VS Usual care	Gui+Ct+Ba+Gs	ns	135	16.0	82.2	NR	24	24	Fatigue, depression, anxiety, physical function
Janse 2018	Netherlands	CDC criteria	Guided self-help CBT VS Waitlist	Gui+Ct+Ba+Gs +Ps+Pr	Wait	240	38.0	60.4	NR	24	NR	Fatigue, quality of life, pain, physical function, depression, anxiety
Lloyd 2012	UK	CDC or Oxford criteria	Individual CBT VS Psychoeducation	Ind+Ct+Ba+Ho +Pr+Ss	Ind+Ba+Pr+Ps	44	15.0	72.7	NR	24	96	Fatigue, physical function
White 2011	UK	Oxford criteria	Individual CBT VS Usual care	Ind+Ct+Ba+Pr	ns	321	38.0	78.2	129	24	48	depression, anxiety, sleep
Chalder 2010	UK	Oxford or CDC criteria	Individual CBT VS Psychoeducation	Ind+Ct+Ba+Ps+ Ho+Pr+Ss	Ind+Pr+Ps+Ba	63	15.0	68.3	96	24	12, 24, 48	Fatigue, physical function, quality of life
Knoop 2008	Netherlands	CDC criteria	Guided self-help CBT VS Waitlist	Gui+Ho+Gs	Wait	169	38.0	79.3	708	16	NR	Fatigue, physical

												function
Rimes 2013	UK	CDC or Oxford criteria	Group CBT VS Waitlist	Gro+Ct+3w	Wait	35	44.0	82.9	346	24	8, 24	Fatigue, physical function, anxiety, depression
Wiborg 2015	Netherlands	CDC criteria	Group CBT VS Waitlist	Gro+Ct+Ba+Gs	Wait	204	38.0	77.0	419	24	NR	Fatigue, physical function
Mohammad 2006	Egypt	CDC criteria	Individual CBT VS Usual care	Ind+Ct+Ba+Ps+Re	ns	92	13.0	72.8	105	72	NR	Fatigue, pain
Gotaas 2021	Norway	CDC or CCC criteria	Individual CBT VS Waitlist	Ind+Ct+Ba+Ps+Ss+Gs	Wait	230	35.0	82.2	230	16	48	Fatigue, quality of life, physical function, pain
Jason 2007	USA	CDC criteria	Individual CBT VS Relaxation	Ind+Ct+Ba+Ho+Pr+Gs	Ind+Ho+Re	114	43.8	83.0	NR	24	48	Fatigue, pain, physical function, anxiety, depression, quality of life
O'Dowd 2006	UK	CDC criteria	Group CBT VS	Gro+Ct+Ba+Gs	ns	153	41.0	66.7	NR	16	8, 32	Fatigue,

			Usual care									depression, physical function, anxiety, cognitive function, quality of life
Prins 2001	Netherlands	CDC criteria	Individual CBT VS Group CBT VS Usual care	Ind+Ct+Ba Gro+Ct+Ba	ns	270	37.0	78.5	269	32	32, 56	Fatigue, quality of life, anxiety, sleep
Sharpe 1996	UK	Oxford criteria	Individual CBT VS Usual care	Ind+Ct+Ba+Pr+Ss	ns	60	36.0	68.3	127	20	20, 32, 48	Fatigue, anxiety, depression, quality of life, physical function
Lloyd 1993	Australia	Lloyd 1988 criteria	Individual CBT VS waitlist	Ind+Ct+Ba+Pr+Gs	ns	90	40.0	75.6	264	16	12	Pain, quality of life, physical function, anxiety, depression,

S2.5 Summary of excluded studies

Lopez 2011[1]	Wrong intervention
Surawy 2005[2]	Wrong intervention
Tummers 2012[3]	Wrong intervention
Rimes 2013[4]	Wrong study design
White 2013[5]	Study duplicate
Gotaas 2023[6]	Wrong study design
Bourke 2014[7]	Wrong study design
Prins 2004[8]	Wrong study design
Cox 2009[9]	Wrong study design
Hall 2017[10]	Wrong study design
Serrat 2024[11]	Wrong intervention
Vos-Vromans 2013[12]	Wrong intervention
Vos-Vromans 2017[13]	Wrong intervention
Hlavaty 2011[14]	Wrong study design
O'Dowd 2006[15]	Wrong study design
Schreurs 2011[16]	Wrong study design
Crawley 2012[17]	Wrong study design
Nijhof 2013[18]	Wrong study design
Tummers 2012[19]	Wrong intervention
Vos-Vromans 2013[20]	Wrong intervention
Vos-Vromans 2017[21]	Wrong intervention
Deale 1996[22]	Study duplicate
Gaunt 2024[23]	Wrong intervention

Windthorst 2017[24]	Wrong intervention
Bazelmans 2002[25]	Wrong study design
Bourke 2014[26]	Wrong study design
Crawley 2024[27]	Wrong study design
Knoop 2013[28]	Wrong study design
Knoop 2008[29]	Wrong study design
Arroll 2012[30]	Wrong study design
Butler 1991[31]	Wrong study design
Kawatani 2011[32]	Wrong study design
Kewley 2011[33]	Wrong study design
Lattie 2016[34]	Wrong study design
Malik 2020[35]	Wrong population
Moss-Morris 2010[36]	Wrong study design
Nijhof 2013[37]	Wrong study design
O'Dowd 2020[38]	Wrong population
Prins 2004[39]	Wrong study design
Saxty 2005[40]	Wrong study design
Stubhaug 2008[41]	Wrong population
White 2015[42]	Wrong study design
Wilshire 2017[43]	Wrong study design
Worm-Smeitink 2019[44]	Wrong study design
Xie 2024[45]	Wrong intervention
Xie 2024[46]	Wrong intervention
Chen 2014[47]	Wrong intervention
Xia 2018[48]	Wrong intervention

Huang 2022[49]	Wrong intervention
Qian 2016[50]	Wrong study design
Su 2024[51]	Wrong study design

1. Lopez C, Antoni M, Penedo F, et al. A pilot study of cognitive behavioral stress management effects on stress, quality of life, and symptoms in persons with chronic fatigue syndrome. Journal of psychosomatic research. 2011;70(4):328-34.
2. Surawy C, Roberts J, Silver A. The Effect of Mindfulness Training on Mood and Measures of Fatigue, Activity, and Quality of Life in Patients with Chronic Fatigue Syndrome on a Hospital Waiting List: A Series of Exploratory Studies. Behavioural and Cognitive Psychotherapy. 2005;33(1):103-9.
3. Tummers M, Knoop H, van Dam A, Bleijenberg G. Implementing a minimal intervention for chronic fatigue syndrome in a mental health centre: a randomized controlled trial. Psychological Medicine. 2012;42(10):2205-15.
4. Rimes KA, Wingrove J. Mindfulness-based cognitive therapy for people with chronic fatigue syndrome still experiencing excessive fatigue after cognitive behaviour therapy: a pilot randomized study. Clinical psychology & psychotherapy. 2013;20(2):107-17.
5. White PD, Goldsmith K, Johnson AL, Chalder T, Sharpe M, Grp PTM. Recovery from chronic fatigue syndrome after treatments given in the PACE trial. Psychological Medicine. 2013;43(10):2227-35.
6. Gotaas ME, Landmark T, Helvik AS, Fors EA. Characteristics associated with physical functioning and fatigue in patients with chronic fatigue syndrome (CFS): secondary analyses of a randomized controlled trial. Fatigue: biomedicine, health and behavior. 2023;11(2-4):66-82.
7. Bourke JH, Johnson AL, Sharpe M, Chalder T, White PD. Pain in chronic fatigue syndrome: response to rehabilitative treatments in the PACE trial. Psychological medicine. 2014;44(7):1545-52.
8. Prins JB, Bos E, Huibers MJH, et al. Social support and the persistence of complaints in chronic fatigue syndrome. Psychotherapy and psychosomatics. 2004;73(3):174-82.
9. Cox DL, Araoz G. The experience of therapy supervision within a UK multi-centre randomized controlled trial. Learning in health & social care. 2009;8(4):301-14.
10. Hall DL, Lattie EG, Milrad SF, et al. Telephone-administered versus live group cognitive behavioral stress management for adults with CFS. Journal of Psychosomatic Research. 2017;93:41-7.
11. Serrat M, Navarrete J, Ferres S, et al. Effectiveness of an online multicomponent program (FATIGUEWALK) for chronic fatigue syndrome: a randomized controlled trial. Health psychology. 2024;43(4):310-22.

12. Vos-Vromans DCWM, Huijnen IPJ, Koke AJA, Seelen HAM, Knottnerus JA, Smeets RJEM. Differences in physical functioning between relatively active and passive patients with Chronic Fatigue Syndrome. Journal of Psychosomatic Research. 2013;75(3):249-54.
13. Vos-Vromans D, Evers S, Huijnen I, et al. Economic evaluation of multidisciplinary rehabilitation treatment versus cognitive behavioural therapy for patients with chronic fatigue syndrome: A randomized controlled trial. Plos One. 2017;12(6):21.
14. Hlavaty LE, Brown MM, Jason LA. The effect of homework compliance on treatment outcomes for participants with myalgic encephalomyelitis/chronic fatigue syndrome. Rehabilitation Psychology. 2011;56(3):212-8.
15. O'Dowd H, Gladwell P, Rogers CA, Hollinghurst S, Gregory A. Cognitive behavioural therapy in chronic fatigue syndrome: a randomised controlled trial of an outpatient group programme. Health technology assessment (Winchester, England). 2006;10(37):iii-121.
16. Schreurs KMG, Veehof MM, Passade L, Vollenbroek-Hutten MMR. Cognitive behavioural treatment for chronic fatigue syndrome in a rehabilitation setting: Effectiveness and predictors of outcome. Behaviour Research and Therapy. 2011;49(12):908-13.
17. Crawley EM. Internet-based cognitive behavioural therapy (FITNET) is an effective treatment for adolescents with chronic fatigue syndrome. Archives of disease in childhood: education and practice edition. 2012;97(6):238.
18. Nijhof SL, Priesterbach LP, Uiterwaal CSPM, Bleijenberg G, Kimpen JLL, van de Putte EM. Internet-based therapy for adolescents with chronic fatigue syndrome: long-term follow-up. Pediatrics. 2013;131(6):e1788-95.
19. Tummers M, Knoop H, van Dam A, Bleijenberg G. Implementing a minimal intervention for chronic fatigue syndrome in a mental health centre: a randomized controlled trial. Psychological medicine. 2012;42(10):2205-15.
20. Vos-Vromans D, Huijnen IPJ, Köke AJA, Seelen HAM, Knottnerus JA, Smeets R. Differences in physical functioning between relatively active and passive patients with Chronic Fatigue Syndrome. Journal of Psychosomatic Research. 2013;75(3):249-54.
21. Vos-Vromans D, Evers S, Huijnen I, et al. Economic evaluation of multidisciplinary rehabilitation treatment versus cognitive behavioural therapy for patients with chronic fatigue syndrome: a randomized controlled trial. PloS one. 2017;12(6):e0177260.
22. Deale A, Chalder T, Marks I, Wessely S. Cognitive behaviour therapy for chronic fatigue syndrome: a randomised controlled trial. South thames research and development, open day, feb 28, 1996. 1996.
23. Gaunt DM, Brigden A, Harris SRS, et al. Graded exercise therapy compared to activity management for paediatric chronic fatigue syndrome/myalgic encephalomyelitis: pragmatic randomized controlled trial. European journal of pediatrics. 2024;183(5):2343-51.
24. Windthorst P, Mazurak N, Kuske M, et al. Heart rate variability biofeedback therapy and graded exercise training in management of chronic fatigue syndrome: An exploratory pilot study. Journal of Psychosomatic Research. 2017;93:6-13.

25. Bazelmans E, Prins J, Bleijenberg G. Cognitive behavior therapy for active and for passive CFS patients. Cognitieve gedragstherapie bij relatief actieve en bij passieve CVS-patienten. 2002;35(2):191-204.
26. Bourke JH, Johnson AL, Sharpe M, Chalder T, White PD. Pain in chronic fatigue syndrome: response to rehabilitative treatments in the PACE trial. Psychological medicine. 2014;44(7):1545-52.
27. Crawley E, Anderson E, Cochrane M, et al. Comparison of cognitive behaviour therapy versus activity management, both delivered remotely, to treat paediatric chronic fatigue syndrome/myalgic encephalomyelitis: the UK FITNET-NHS RCT. Health technology assessment (Winchester, England). 2024;28(70):1-134.
28. Knoop H, Heins M, Bloot L, Wiborg. A treatment model for cognitive-behavioural interventions for chronic fatigue syndrome: Work in progress. Journal of Psychosomatic Research. 2013;74(6):550.
29. Knoop H, Stulemeijer M, de Jong L, Fiselier TJW, Bleijenberg G. Efficacy of cognitive behavioral therapy for adolescents with chronic fatigue syndrome: Long-term follow-up of a randomized, controlled trial. Pediatrics. 2008;121(3):E619-E25.
30. Arroll MA, Howard A. A preliminary prospective study of nutritional, psychological and combined therapies for myalgic encephalomyelitis/chronic fatigue syndrome (ME/CFS) in a private care setting. Bmj Open. 2012;2(6):12.
31. Butler S, Chalder T, Ron M, Wessely S. Cognitive behaviour therapy in chronic fatigue syndrome. J Neurol Neurosurg Psychiatry. 1991;54(2):153-8.
32. Kawatani J, Mizuno K, Shiraishi S, et al. Cognitive dysfunction and mental fatigue in childhood chronic fatigue syndrom-A 6-month follow-up study. Brain & Development. 2011;33(10):832-41.
33. Kewley AJ. The PACE trial in chronic fatigue syndrome. Lancet (london, england). 2011;377(9780):1832; author reply 4-5.
34. Lattie EG. The effects of telephone-delivered cognitive behavioral stress management on inflammation and symptoms in Myalgic Encephalomyelitis/Chronic Fatigue Syndrome: A computational immunology approach. Dissertation Abstracts International: Section B: The Sciences and Engineering. 2016;77(1-B(E)):No-Specified.
35. Malik S, Asprusten TT, Pedersen M, et al. Cognitive-behavioural therapy combined with music therapy for chronic fatigue following Epstein-Barr virus infection in adolescents: a randomised controlled trial. BMJ paediatrics open. 2020;4(1):e000797.
36. Moss-Morris R, Hamilton W. Pragmatic rehabilitation for chronic fatigue syndrome. BMJ-British Medical Journal. 2010;340:3.
37. Nijhof SL, Priesterbach LP, Uiterwaal C, Bleijenberg G, Kimpen JLL, van de Putte EM. Internet-Based Therapy for Adolescents With Chronic Fatigue Syndrome: Long-term Follow-up. Pediatrics. 2013;131(6):E1788-E95.
38. O'Dowd H, Beasant L, Ingram J, et al. The feasibility and acceptability of an early intervention in primary care to prevent chronic fatigue syndrome (CFS) in adults: randomised controlled trial. Pilot and feasibility studies. 2020;6(1):65.

39. Prins JB, Bos E, Huibers MJH, et al. Social support and the persistence of complaints in chronic fatigue syndrome. Psychotherapy and Psychosomatics. 2004;73(3):174-82.
40. Saxty M, Hansen Z. Group cognitive behavioural therapy for chronic fatigue syndrome: A pilot study. Behavioural and Cognitive Psychotherapy. 2005;33(3):311-8.
41. Stubhaug B, Lie SA, Ursin H, Eriksen HR. Cognitive-behavioural therapy v. mirtazapine for chronic fatigue and neurasthenia: randomised placebo-controlled trial. British journal of psychiatry. 2008;192(3):217-23.
42. White PD, Chalder T, Sharpe M. The planning, implementation and publication of a complex intervention trial for chronic fatigue syndrome: The PACE trial. BJPsych Bulletin. 2015;39(1):24-7.
43. Wilshire C, Kindlon T, Matthees A, McGrath S. Can patients with chronic fatigue syndrome really recover after graded exercise or cognitive behavioural therapy? A critical commentary and preliminary re-analysis of the PACE trial. Fatigue: Biomedicine, Health and Behavior. 2017;5(1):43-56.
44. Worm-Smeitink M, Janse A, van Dam A, et al. Internet-Based Cognitive Behavioral Therapy in Stepped Care for Chronic Fatigue Syndrome: randomized Noninferiority Trial. Journal of medical Internet research. 2019;21(3):e11276.
45. XIE F F, GU Y J, GUAN C, XIE C Q, XU J T, YAO F. The application value of fractional amplitude of low frequency fluctuation in the treatment of chronic fatigue syndrome with anxiety depression by Prolong Life With Nine Turn method[J]. Chin J Magn Reson Imaging, 2024, 15(7): 58-63.
46. Xie F, Xie C, Yue H, et al. Study on resting state functional magnetic resonance imaging in intervention of sleep disorder in chronic fatigue syndrome by prolong life with nine turn method (Yan Nian Jiu Zhuan) Qigong. J Clin Radiol. 2024;43(6):1009-1016.
47. Chen ZR, Liu TR. The clinical efficacy of the Chinese herbal paste combined with auricular-plaster therapy on sleep disturbance in nurses with chronic fatigue syndrome. J Nurs Sci. 2014;29(13):13-15.
48. Xia ML, Xia MF, Wu YJ, Liu XT. Application of head-scraping combined with five-sound therapy in chronic fatigue syndrome of liver depression and qi stagnation type [J]. J Clin Pathol Res, 2018, 36(6): 1252-1257. doi: 10.3978/j.issn.2095-6959.2018.06.025.
49. Huang Q, Zhang ZX, Li GW, Chen C, Zhi JN, Chen M, Zhang WJ. The effect of Wen Zhen Yun Qi formula on brain function and metabolism in patients with chronic fatigue syndrome and Qi deficiency syndrome [J]. J Tradit Chin Med, 2022, 63(10): 943-950. DOI: 10.13288/j.11-2166kc.2022.10.010.
50. Qian LF, Yao Q, Zhu GF. Effect of channel points massage combined with Yijinjing exercising on chronic fatigue syndrome with sleep disorder. Chin J Gen Pract. 2016;14(11):1886-1889. doi:10.16766/j.cnki.issn.1674-4152.2016.11.030
51. Su JN, Wang XY, Dan YQ, et al. Effects of therapy of "five tones regulating the spirit" on chronic fatigue syndrome with anxiety based on the "brain-gut axis". Mod J Integr Tradit Chin West Med. 2024;33(13):1765-1771. doi:10.3969/j.issn.1008-8849.2024.13.003

Appendix S3: Risk of bias assessments

Study ID	Outcome	Result	Randomization process	Deviations from intended interventions	Missing outcome data	Measurement of the outcome	Selection of the reported result	Overall Bias
Chalder 2010	Fatigue	Chalder Fatigue Scale	Low	Some concerns	Low	Low	Some concerns	Some concerns
Chalder 2010	Physical function	SF-36 Physical function	Low	Some concerns	Low	Low	Some concerns	Some concerns
Deale 1997	Fatigue	Chalder Fatigue Scale	Low	Low	Low	Low	Some concerns	Some concerns
Deale 1997	Depression	BDI	Low	Low	Low	Low	Some concerns	Some concerns
Deale 1997	Physical function	SF-36 Physical function	Low	Low	Low	Low	Some concerns	Some concerns
Gotaas 2021	Fatigue	Chalder Fatigue Scale	Low	Low	Low	Low	Low	Low
Gotaas 2021	Anxiety	HADS	Low	Low	Low	Low	Low	Low
Gotaas 2021	Depression	HADS	Low	Low	Low	Low	Low	Low

Gotaas 2021	Physical function	SF-36	Low	Low	Low	Low	Low	Low
Janse 2017	Fatigue	CIS	Low	Low	Low	Low	Low	Low
Janse 2017	Depression	SCL-90	Low	Low	Low	Low	Low	Low
Janse 2017	Physical function	SF-36 Physical function	Low	Low	Low	Low	Low	Low
Knoop 2008	Fatigue	CIS	Low	Low	Low	Low	Low	Low
Knoop 2008	Physical function	SF–36 Physical function	Low	Low	Low	Low	Low	Low
Lloyd 2012	Fatigue	Chalder Fatigue Scale	Low	Some concerns	Low	Low	Some concerns	Some concerns
Lloyd 2012	Physical function	SF-36 Physical function	Low	Some concerns	Low	Low	Some concerns	Some concerns
McDermott 2006	Fatigue	Chalder Fatigue Scale	Low	Low	Low	Low	Some concerns	Some concerns
McDermott 2006	Quality of life	WHOQOL-B REF	Low	Low	Low	Low	Some concerns	Some concerns
McDermott 2006	Anxiety	HADS	Low	Low	Low	Low	Some concerns	Some concerns

McDermott 2006	Depression	HADS	Low	Low	Low	Low	Some concerns	Some concerns
Mohammad 2006	Fatigue	CIS	Some concerns	Some concerns	Some concerns	Low	Some concerns	Some concerns
Nijhof 2012	Fatigue	CIS	Low	Low	Low	Low	Low	Low
Nijhof 2012	Physical function	CHQ	Low	Low	Low	Low	Low	Low
O'Dowd 2006	Fatigue	Chalder Fatigue Scale	Low	Low	Low	Low	Some concerns	Some concerns
O'Dowd 2006	Quality of life	SF-36	Low	Low	Low	Low	Some concerns	Some concerns
O'Dowd 2006	Anxiety	HADS	Low	Low	Low	Low	Some concerns	Some concerns
O'Dowd 2006	Depression	HADS	Low	Low	Low	Low	Some concerns	Some concerns
O'Dowd 2006	Physical function	SF-36 Physical function	Low	Low	Low	Low	Some concerns	Some concerns
Rimes 2013	Fatigue	Chalder Fatigue Scale	Low	Low	Low	Low	Some concerns	Some concerns
Rimes 2013	Anxiety	HADS	Low	Low	Low	Low	Some concerns	Some concerns
Rimes 2013	Depression	HADS	Low	Low	Low	Low	Some concerns	Some concerns

Rimes 2013	Physical function	SF-36 Physical function	Low	Low	Low	Low	Some concerns	Some concerns
Stulemeijer 2005	Fatigue	CIS	Low	Low	Low	Low	Some concerns	Some concerns
Stulemeijer 2005	Physical function	SF-36 Physical function	Low	Low	Low	Low	Some concerns	Some concerns
White 2011	Fatigue	Chalder Fatigue Scale	Low	Low	Low	Low	Low	Low
White 2011	Sleep	Jenkins sleep scale	Low	Low	Low	Low	Low	Low
White 2011	Anxiety	HADS	Low	Low	Low	Low	Low	Low
White 2011	Depression	HADS	Low	Low	Low	Low	Low	Low
Wiborg 2015	Fatigue	CIS	Low	Low	High	Low	Some concerns	High
Sharpe 2015	Fatigue	Chalder Fatigue Scale	Low	Low	Low	Low	Low	Low
Sharpe 2015	Physical function	SF-36 Physical function	Low	Low	Low	Low	Low	Low
Burgess 2012	Fatigue	Chalder Fatigue Scale	Some concerns	Low	Low	Low	Some concerns	Some concerns
Burgess 2012	Physical function	Medical Outcomes Survey	Some concerns	Low	Low	Low	Some concerns	Some concerns

Prins 2001	quality of life	sickness impact profile	Low	Low	Low	Low	Low	Low
Sharpe 1996	anxiety	HADS anxiety score	Low	Low	Low	Low	Some concerns	Some concerns
Sharpe 1996	depression	HADS depression score	Low	Low	Low	Low	Some concerns	Some concerns
Lloyd 1993	quality of life	VAS	Low	Low	Low	Low	Some concerns	Some concerns
Lloyd 1993	anxiety	Profile of Mood States	Low	Low	Low	Low	Some concerns	Some concerns
Lloyd 1993	depression	Profile of Mood States	Low	Low	Low	Low	Some concerns	Some concerns
Lloyd 1993	Physical function	Karnofsky Performance Scale	Low	Low	Low	Low	Some concerns	Some concerns

Abbreviations: CIS = Checklist Individual Strength, SF-36 = Short Form 36-Questionnaire, BDI = Beck Depression Inventory, HADS = Hospital Anxiety and Depression Scale, WHOQOL = Modified World Health Organization Quality of life questionnaire, SCL-90 = Symptom Checklist-90, VAS = Visual analog scales, CHQ = Child Health Questionnaire.

Appendix S4 Main results at treatment level

Appendix S4.1 Network estimates (league tables)

The league tables show the relative effects of each intervention compared to minimal intervention (the column's treatment versus the row's treatment). The relative effects are measured as a mean difference for outcomes (S5.2.1- S5.2.8) along with 95% CIs. The colour of each cell indicates the certainty of evidence according, the Grading of Recommendations Assessment, Development, and Evaluation (GRADE).

Figure Legend

Certainty of evidence

High Moderate Low Very low

S4.1.1 Fatigue when measured immediately after treatments

Group					
-0.58(-4.65 to 3.49)	Guided self-help				
-2.66(-6.41 to 1.10)	-2.08(-5.34 to 1.18)	Individual			
-4.36(-11.50 to 2.79)	-3.78(-10.68 to 3.12)	-1.70(-7.78 to 4.38)	Psychoeducation		
-3.56(-11.92 to 4.81)	-2.98(-11.13 to 5.17)	-0.90(-8.37 to 6.57)	0.80(-8.84 to 10.44)	Relaxation	
-7.47(-10.54 to -4.39)	-6.89(-9.56 to -4.22)	-4.81(-6.98 to -2.65)	-3.11(-9.57 to 3.34)	-3.91(-11.69 to 3.87)	Minimal intervention

S4.1.2 Physical function when measured immediately after treatments

Group					
0.46(-9.55 to 10.47)	Guided self-help				
3.56(-6.34 to 13.46)	3.10(-4.35 to 10.54)	Individual			
5.56(-15.90 to 27.02)	5.10(-15.35 to 25.54)	2.00(-17.04 to 21.04)	Psychoeducation		
25.16(5.79 to 44.53)	24.70(6.46 to 42.93)	21.60(4.95 to 38.25)	19.60(-5.69 to 44.89)	Relaxation	
11.33(3.24 to 19.41)	10.87(4.96 to 16.77)	7.77(2.04 to 13.49)	5.77(-14.12 to 25.65)	-13.83(-31.44 to 3.77)	Minimal intervention

S4.1.3 Fatigue at the end of the follow-up

Group					
-4.17(-15.55 to 7.21)	Guided self-help				
-0.99(-7.87 to 5.89)	3.18(-5.88 to 12.24)	Individual			
-2.74(-11.80 to 6.32)	1.43(-9.38 to 12.24)	-1.75(-7.64 to 4.14)	Psychoeducation		
-5.34(-13.74 to 3.07)	-1.17(-11.43 to 9.10)	-4.35(-9.17 to 0.48)	-2.60(-10.21 to 5.02)	Relaxation	
-2.79(-6.97 to 1.39)	1.38(-9.21 to 11.97)	-1.80(-7.27 to 3.67)	-0.05(-8.09 to 7.99)	2.55(-4.75 to 9.84)	Minimal intervention

S4.1.4 Physical function at the end of the follow-up

Group					
1.39(-83.47 to 86.25)	Guided self-help				
4.90(-64.18 to 73.98)	3.51(-45.77 to 52.79)	Individual			
10.49(-74.88 to 95.86)	9.10(-61.22 to 79.42)	5.59(-44.57 to 55.75)	Psychoeducation		
20.51(-56.95 to 97.97)	19.12(-41.34 to 79.58)	15.61(-19.43 to 50.65)	10.02(-51.17 to 71.21)	Relaxation	
9.70(-40.29 to 59.69)	8.31(-60.26 to 76.88)	4.80(-42.88 to 52.48)	-0.79(-69.99 to 68.41)	-10.81(-69.98 to 48.36)	Minimal intervention

S4.1.5 Depression at the end of the follow-up

Group			
-0.11(-1.71 to 1.48)	Individual		
-0.62(-2.57 to 1.34)	-0.50(-1.63 to 0.62)	Relaxation	
-1.11(-2.38 to 0.16)	-1.00(-1.96 to -0.04）	-0.50(-1.98 to 0.99)	Minimal intervention

Appendix S4.2 Forest plots

S4.2.1 Fatigue when measured immediately after treatments

When the number of studies for a comparison is 0, this indicates the absence of direct head-to-head evidence. In such cases, an effect estimate may still be shown in the forest plot because it is derived via the network. When no direct evidence is available, the overall network estimate relies entirely on, and is identical to, the indirect estimate.

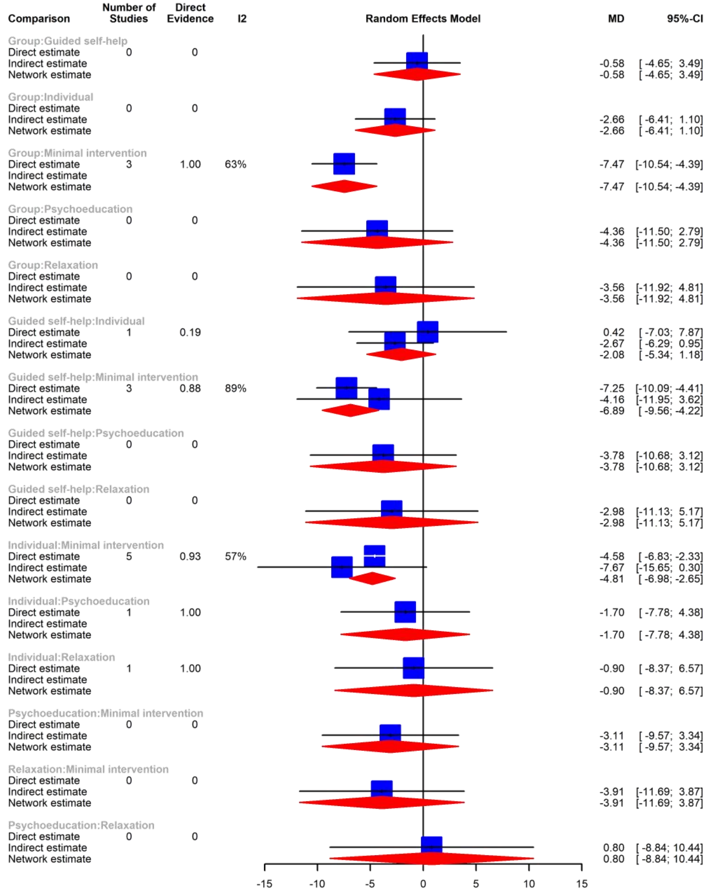

S4.2.2 Physical function when measured immediately after treatments

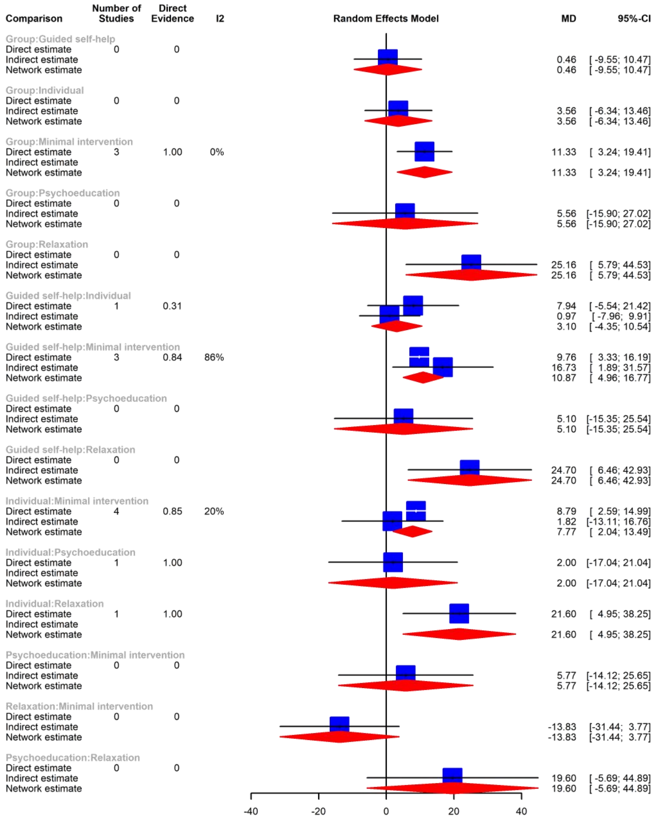

S4.2.3 Fatigue at the end of the follow-up

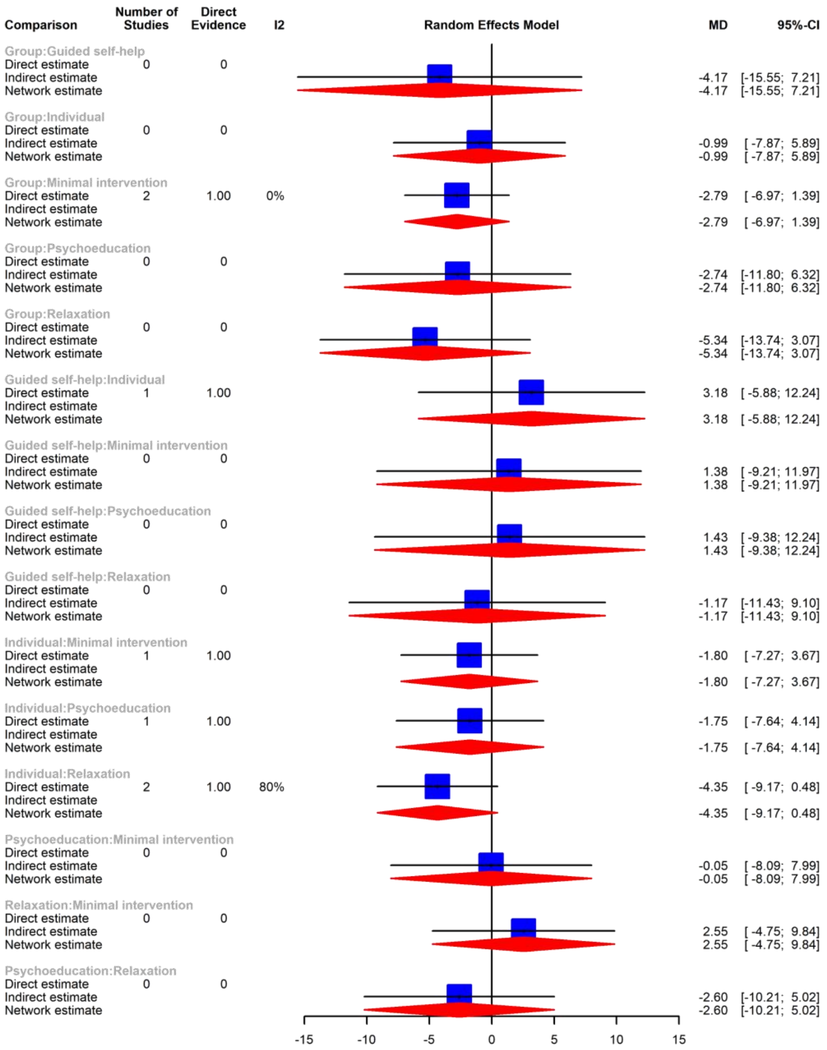

S4.2.4 Physical function at the end of the follow-up

Comparison	Number of Studies	Direct Evidence	I2	MD	95%-CI
**Group:Guided self-help**					
Direct estimate	0	0			
Indirect estimate				1.39	[-83.47; 86.25]
Network estimate				1.39	[-83.47; 86.25]
**Group:Individual**					
Direct estimate	0	0			
Indirect estimate				4.90	[-64.18; 73.98]
Network estimate				4.90	[-64.18; 73.98]
**Group:Minimal intervention**					
Direct estimate	1	1.00		9.70	[-40.29; 59.69]
Indirect estimate					
Network estimate				9.70	[-40.29; 59.69]
**Group:Psychoeducation**					
Direct estimate	0	0			
Indirect estimate				10.49	[-74.88; 95.86]
Network estimate				10.49	[-74.88; 95.86]
**Group:Relaxation**					
Direct estimate	0	0			
Indirect estimate				20.51	[-56.95; 97.97]
Network estimate				20.51	[-56.95; 97.97]
**Guided self-help:Individual**					
Direct estimate	1	1.00		3.51	[-45.77; 52.79]
Indirect estimate					
Network estimate				3.51	[-45.77; 52.79]
**Guided self-help:Minimal intervention**					
Direct estimate	0	0			
Indirect estimate				8.31	[-60.26; 76.88]
Network estimate				8.31	[-60.26; 76.88]
**Guided self-help:Psychoeducation**					
Direct estimate	0	0			
Indirect estimate				9.10	[-61.22; 79.42]
Network estimate				9.10	[-61.22; 79.42]
**Guided self-help:Relaxation**					
Direct estimate	0	0			
Indirect estimate				19.12	[-41.34; 79.58]
Network estimate				19.12	[-41.34; 79.58]
**Individual:Minimal intervention**					
Direct estimate	1	1.00		4.80	[-42.88; 52.48]
Indirect estimate					
Network estimate				4.80	[-42.88; 52.48]
**Individual:Psychoeducation**					
Direct estimate	1	1.00		5.59	[-44.57; 55.75]
Indirect estimate					
Network estimate				5.59	[-44.57; 55.75]
**Individual:Relaxation**					
Direct estimate	2	1.00	91%	15.61	[-19.43; 50.65]
Indirect estimate					
Network estimate				15.61	[-19.43; 50.65]
**Psychoeducation:Minimal intervention**					
Direct estimate	0	0			
Indirect estimate				-0.79	[-69.99; 68.41]
Network estimate				-0.79	[-69.99; 68.41]
**Relaxation:Minimal intervention**					
Direct estimate	0	0			
Indirect estimate				-10.81	[-69.98; 48.36]
Network estimate				-10.81	[-69.98; 48.36]
**Psychoeducation:Relaxation**					
Direct estimate	0	0			
Indirect estimate				10.02	[-51.17; 71.21]
Network estimate				10.02	[-51.17; 71.21]

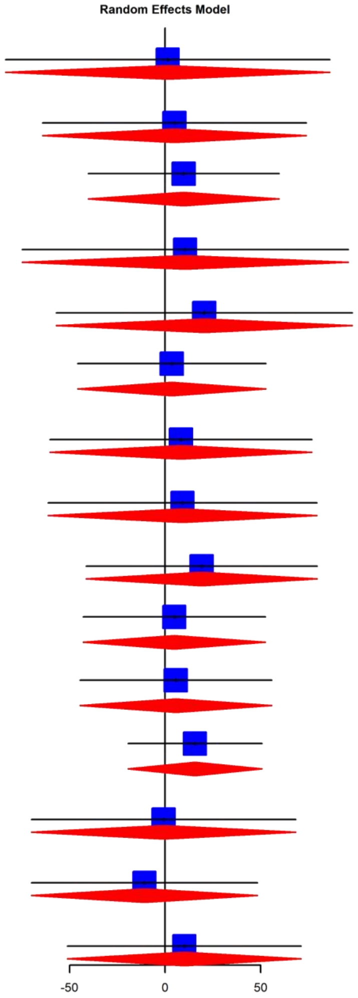

S4.2.5 Depression at the end of the follow-up

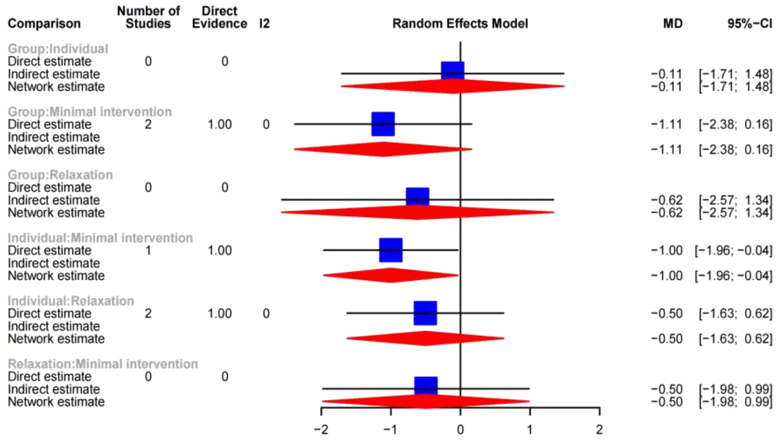

Appendix S4.3 Heterogeneity

S4.3.1 Fatigue when measured immediately after treatments

Study	Experimental Total	Experimental Mean	Experimental SD	Control Total	Control Mean	Control SD	MD	95%-CI	Weight
subgroup = A D									
Rimes2013	16	21.30	6.2000	19	25.20	4.7000	-3.90	[-7.60; -0.20]	6.8%
Wiborg2015a	68	17.67	9.4900	34	26.54	5.8400	-8.87	[-11.86; -5.88]	7.6%
Wiborg2015b	68	17.46	9.2800	34	26.54	5.8400	-9.08	[-12.03; -6.13]	7.6%
Random effects model	152			87			-7.46	[-10.59; -4.33]	21.9%
Heterogeneity: $I^2 = 63\%$, $\tau^2 = 4.9880$, $p = 0.06$									
subgroup = B C									
Burgess2012	29	22.65	12.5700	29	22.23	11.1900	0.42	[-5.71; 6.55]	4.4%
subgroup = B D									
Janse2018	160	19.70	9.5100	80	24.68	7.2200	-4.98	[-7.14; -2.82]	8.4%
Knoop2008	84	21.24	8.3200	85	26.40	5.9800	-5.16	[-7.35; -2.97]	8.4%
Nijhof2012	67	11.00	9.2100	64	23.58	9.0100	-12.58	[-15.70; -9.46]	7.4%
Random effects model	311			229			-7.45	[-12.24; -2.67]	24.3%
Heterogeneity: $I^2 = 89\%$, $\tau^2 = 16.2536$, $p < 0.01$									
subgroup = C D									
Gotaas2021a	76	18.70	7.3400	39	24.10	6.9800	-5.40	[-8.14; -2.66]	7.8%
Gotaas2021b	76	20.70	7.3400	39	24.10	6.9800	-3.40	[-6.14; -0.66]	7.8%
Mohammad2006	50	10.63	1.2500	42	15.35	4.6900	-4.72	[-6.18; -3.26]	9.1%
Sharpe2015	148	21.50	7.8000	152	24.00	6.9000	-2.50	[-4.17; -0.83]	8.9%
Stulemeijer2005	29	15.26	11.5500	33	24.75	9.2100	-9.49	[-14.74; -4.24]	5.2%
Random effects model	379			305			-4.33	[-5.85; -2.80]	38.9%
Heterogeneity: $I^2 = 57\%$, $\tau^2 = 1.4583$, $p = 0.05$									
subgroup = C E									
Chalder2010	29	13.50	8.2000	27	15.20	8.4000	-1.70	[-6.05; 2.65]	6.1%
subgroup = C F									
Deale1997	30	21.60	12.0000	30	22.50	12.3000	-0.90	[-7.05; 5.25]	4.4%
**Random effects model**	**930**			**707**			**-5.37**	**[-7.12; -3.62]**	**100.0%**

Heterogeneity: $I^2 = 77\%$, $\tau^2 = 8.2456$, $p < 0.01$
Test for subgroup differences: $\chi^2_5 = 10.32$, df = 5 ($p = 0.07$)

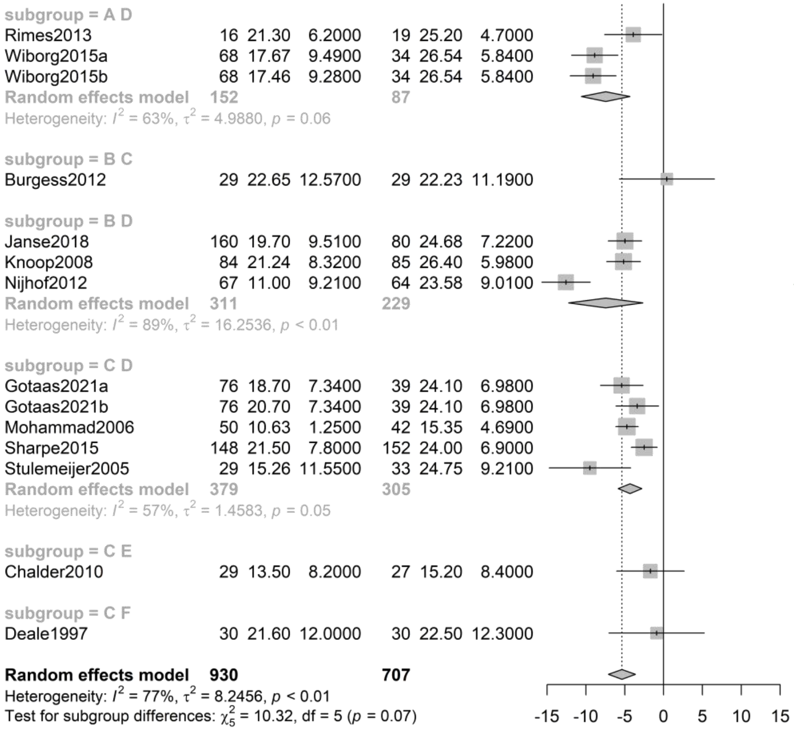

S4.3.2 Physical function when measured immediately after treatments

Study	Experimental Total	Experimental Mean	Experimental SD	Control Total	Control Mean	Control SD	MD	95%−CI	Weight
**subgroup = A D**									
Rimes2013	16	65.90	23.1000	19	53.70	23.1000	12.20	[−3.16; 27.56]	3.6%
Wiborg2015a	68	77.40	20.6000	34	63.30	21.1000	14.10	[5.48; 22.72]	7.9%
Wiborg2015b	68	71.30	23.0000	34	63.30	21.1000	8.00	[−0.95; 16.95]	7.6%
**Random effects model**	**152**			**87**			**11.31**	**[5.55; 17.07]**	**19.1%**
Heterogeneity: $I^2 = 0\%$, $\tau^2 = 0$, $p = 0.62$									
**subgroup = B C**									
Burgess2012	29	66.47	17.1400	29	58.53	20.5900	7.94	[−1.81; 17.69]	6.9%
**subgroup = B D**									
Janse2018	160	75.15	23.7100	80	70.80	21.0000	4.35	[−1.54; 10.24]	11.2%
Knoop2008	84	65.90	23.2000	85	60.20	23.7000	5.70	[−1.37; 12.77]	9.6%
Nijhof2012	67	88.50	13.8000	64	70.10	17.6000	18.40	[12.97; 23.83]	11.8%
**Random effects model**	**311**			**229**			**9.63**	**[0.68; 18.58]**	**32.6%**
Heterogeneity: $I^2 = 86\%$, $\tau^2 = 52.7434$, $p < 0.01$									
**subgroup = C D**									
Gotaas2021a	76	71.20	21.7900	39	57.90	20.9500	13.30	[5.10; 21.50]	8.4%
Gotaas2021b	76	62.90	22.0200	39	57.90	20.9500	5.00	[−3.23; 13.23]	8.3%
Sharpe2015	148	54.20	21.6000	152	48.40	23.1000	5.80	[0.74; 10.86]	12.3%
Stulemeijer2005	29	69.40	28.0000	33	55.30	21.1000	14.10	[1.62; 26.58]	5.0%
**Random effects model**	**329**			**263**			**8.20**	**[3.88; 12.53]**	**33.9%**
Heterogeneity: $I^2 = 20\%$, $\tau^2 = 4.2707$, $p = 0.29$									
**subgroup = C E**									
Chalder2010	28	59.40	28.4000	25	57.40	32.8000	2.00	[−14.61; 18.61]	3.2%
**subgroup = C F**									
Deale1997	30	56.20	26.2000	30	34.60	28.3000	21.60	[7.80; 35.40]	4.3%
**Random effects model**	**879**			**663**			**9.84**	**[6.56; 13.13]**	**100.0%**

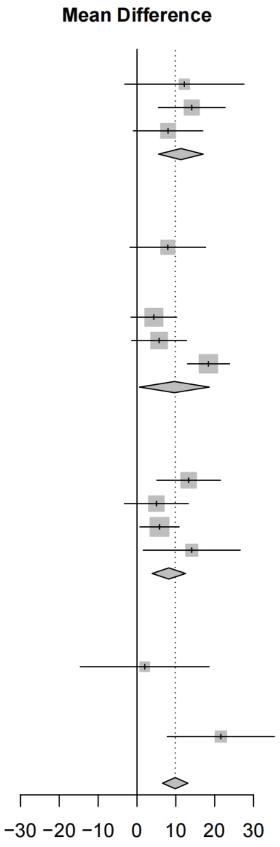

Heterogeneity: $I^2 = 50\%$, $\tau^2 = 16.1670$, $p = 0.02$
Test for subgroup differences: $\chi^2_5 = 4.56$, df = 5 ($p = 0.47$)

S4.3.3 Fatigue at the end of the follow-up

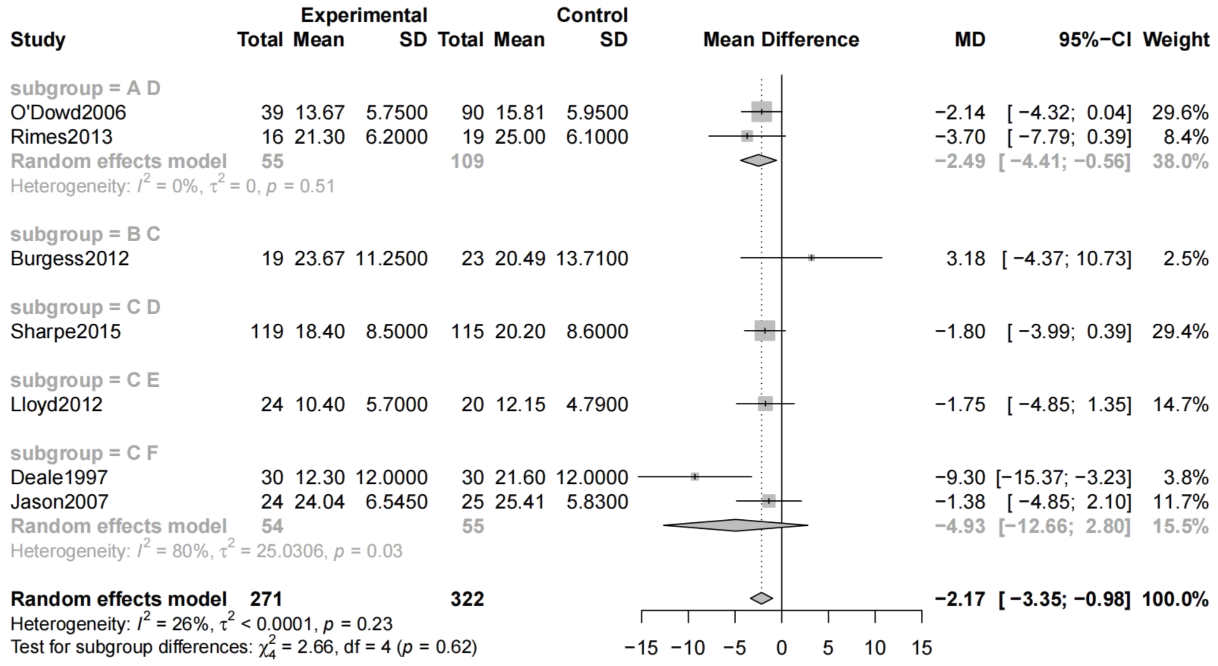

S4.3.4 Physical fnction at the end of the follow-up

Study	Experimental Total	Experimental Mean	Experimental SD	Control Total	Control Mean	Control SD	MD	95%−CI	Weight
subgroup = A D									
Rimes2013	16	65.60	26.3000	19	55.90	23.3000	9.70	[−6.91; 26.31]	14.8%
subgroup = B C									
Burgess2012	18	65.83	21.7300	23	62.32	24.9600	3.51	[−10.80; 17.82]	16.6%
subgroup = C D									
Sharpe2015	119	62.20	27.2000	115	57.40	27.9000	4.80	[−2.26; 11.86]	22.3%
subgroup = C E									
Lloyd2012	24	76.79	29.8100	20	71.20	27.9900	5.59	[−11.52; 22.70]	14.5%
subgroup = C F									
Deale1997	30	71.60	28.0000	30	38.40	26.9000	33.20	[19.31; 47.09]	16.9%
Jason2007	23	58.64	30.4400	25	61.20	27.7000	−2.56	[−19.07; 13.95]	14.9%
Random effects model	53			55			15.61	[−19.43; 50.65]	31.8%
Heterogeneity: $I^2 = 91\%$, $\tau^2 = 578.7728$, $p < 0.01$									
**Random effects model**	**230**			**232**			**9.13**	**[−0.89; 19.15]**	**100.0%**
Heterogeneity: $I^2 = 68\%$, $\tau^2 = 104.3425$, $p < 0.01$									
Test for subgroup differences: $\chi^2_4 = 0.68$, df = 4 ($p = 0.95$)									

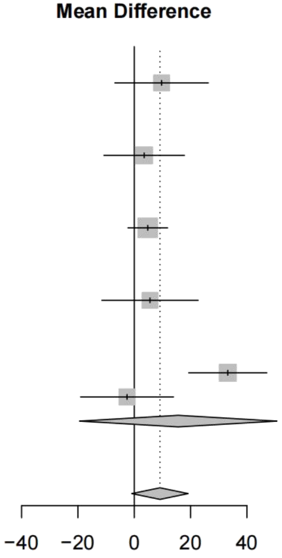

S4.3.5 Depression at the end of the follow-up

Study	Experimental Total	Experimental Mean	Experimental SD	Control Total	Control Mean	Control SD	MD	95%-CI	Weight
**subgroup = A C**									
O'Dowd2006	39	6.82	3.8000	90	7.59	4.2000	-0.77	[-2.24; 0.70]	18.5%
Rimes2013	16	5.60	2.9000	19	7.70	4.6000	-2.10	[-4.61; 0.41]	6.4%
**Random effects model**	**55**			**109**			**-1.11**	**[-2.38; 0.16]**	**24.9%**
Heterogeneity: $I^2 = 0\%$, $\tau^2 = 0$, $p = 0.37$									
**subgroup = B C**									
White2011	143	6.20	3.7000	151	7.20	4.7000	-1.00	[-1.96; -0.04]	43.3%
**subgroup = B D**									
Deale1997	30	3.37	2.3000	30	4.10	2.8300	-0.73	[-2.03; 0.57]	23.6%
Jason2007	23	4.65	4.3600	24	4.50	3.3200	0.15	[-2.07; 2.37]	8.2%
**Random effects model**	**53**			**54**			**-0.50**	**[-1.63; 0.62]**	**31.8%**
Heterogeneity: $I^2 = 0\%$, $\tau^2 = 0$, $p = 0.50$									
**Random effects model**	**251**			**314**			**-0.87**	**[-1.50; -0.24]**	**100.0%**

Heterogeneity: $I^2 = 0\%$, $\tau^2 = 0$, $p = 0.76$

Test for subgroup differences: $\chi^2_2 = 0.61$, df = 2 ($p = 0.74$)

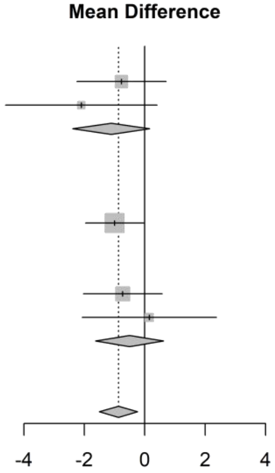

Appendix S4.4 Publication bias (Begger or Egger's test)

S4.4.1 Fatigue when measured immediately after treatments

Comparison	Begg's Test (*z*)	Begg's Test (*p*)	Egger's test (*z*)	Egger's test (*p*)
Group CBT v Minimal intervention	1.04	0.296	-133.37	0.005
Guided self-help CBT v Minimal intervention	1.04	0.296	268.81	0.002
Individual CBTv Minimal intervention	0.51	0.613	-1.10	0.351

S4.4.2 Physical function when measured immediately after treatments

Comparison	Begg's Test (*z*)	Begg's Test (*p*)	Egger's test (*z*)	Egger's test (*p*)
Group CBT vs Minimal intervention	0.00	1.000	0.86	0.549
Guided self-help CBT vs Minimal intervention	0.00	1.000	1.11	0.468
Individual CBT vs Minimal intervention	0.34	0.734	0.04	0.974

S4.4.3 Fatigue at the end of the follow-up

**Comparison**	**Begg's Test (*z*)**	**Begg's Test (*p*)**	**Egger's test (*z*)**	**Egger's test (*p*)**
Group CBT vs Minimal intervention	0.00	1.000	-	-
Individual CBT vs Relaxation	0.00	1.000	-	-

S4.4.4 Physical function at the end of the follow-up

**Comparison**	**Begg's Test (*z*)**	**Begg's Test (*p*)**	**Egger's test (*z*)**	**Egger's test (*p*)**
Individual CBT vs Relaxation	0.00	1.000	-	-

S4.4.5 Depression at the end of the follow-up

**Comparison**	**Begg's Test (*z*)**	**Begg's Test (*p*)**	**Egger's test (*z*)**	**Egger's test (*p*)**
Group CBT vs Minimal intervention	0.00	1.000	-	-
Individual CBT vs Relaxation	0.00	1.000	-	-

Appendix S4.5: GRADE assessments: certainty of evidence for direct, indirect and network estimates

S4.5.1 Fatigue when measured immediately after treatments

Comparisons	Direct	RoB	Indirectness	Inconsistency	Publication Bias	Direct Certainty [a]	Indirect	Indirect Certainty [a]	NMA	Direct + Indirect Certainty [a]	Incoherence	Imprecision	NMA Certainty
Group CBT VS Guided self-help CBT	——	——	——	——	——	——	-0.58(-4.65 to 3.49)	ADDB Moderate	-0.58(-4.65 to 3.49)	Moderate	NA	Very serious	Very low
Group CBT VS Individual CBT	——	——	——	——	——	——	-2.66(-6.41 to 1.10)	ADDC Moderate	-2.66(-6.41 to 1.10)	Moderate	NA	Serious	Low
Group CBT VS Minimal intervention	-7.46(-10.59 to -4.33)	Serious	Not serious	Not serious	Not serious	Moderate	NA	NA	-7.47(-10.54 to -4.39)	Moderate	Not serious	Serious	Low
Group CBT VS Psychoeduca	——	——	——	——	——	——	-4.36(-11.50 to 2.79)	ADCE Moderate	-4.36(-11.50 to 2.79)	Moderate	NA	Very serious	Very low

tion													
Group CBT VS Relaxation	——	——	——	——	——	——	-3.56(-11.92 to 4.81)	ADCF Moderate	-3.56(-11.92 to 4.81)	Moderate	NA	Very serious	Very low
Guided self-help CBT VS Individual	0.42(-5.71 to 6.55)	Serious	Not serious	Not serious	Not serious	Moderate	-2.67(-6.29 to 0.95)	BDDC Moderate	-2.08(-5.34 to 1.18)	Moderate	Not serious	Serious	Low
Guided self-help CBT VS Minimal intervention	-7.45(-12.24 to -2.67)	Not serious	Serious	Not serious	Not serious	Moderate	-4.16(-11.95 to 3.62)	BCCD Moderate	-6.89(-9.56 to -4.22)	Moderate	Not serious	Not serious	Moderate
Guided self-help CBT VS Psychoeducation	——	——	——	——	——	——	-3.78(-10.68 to 3.12)	BCCE Moderate	-3.78(-10.68 to 3.12)	Moderate	NA	Very serious	Very low
Guided self-help CBT VS Relaxation	——	——	——	——	——	——	-2.98(-11.13 to 5.17)	BCCF Moderate	-2.98(-11.13 to 5.17)	Moderate	NA	Very serious	Very low

Individual CBT VS Minimal intervention	-4.33(-5.85 to -2.80)	Not serious	Serious	Not serious	Not serious	Moderate	-7.67(-15.65 to 0.30)	CBBD Moderate	-4.81(-6.98 to -2.65)	Moderate	Not serious	Not serious	Moderate
Individual CBT VS Psychoeducation	-1.70(-6.05 to 2.65)	Serious	Not serious	Not serious	Not serious	Moderate	NA	NA	-1.70(-7.78 to 4.38)	Moderate	Not serious	Very serious	Very low
Individual CBT VS Relaxation	-0.90(-7.05 to 5.25)	Serious	Not serious	Not serious	Not serious	Moderate	NA	NA	-0.90(-8.37 to 6.57)	Moderate	Not serious	Very serious	Very low
Psychoeducation VS Minimal intervention	——	——	——	——	——	——	-3.11(-9.57 to 3.34)	DCCE Moderate	-3.11(-9.57 to 3.34)	Moderate	NA	Very serious	Very low
Relaxation VS Minimal intervention	——	——	——	——	——	——	-3.91(-11.69 to 3.87)	DCCF Moderate	-3.91(-11.69 to 3.87)	Moderate	NA	Very serious	Very low
Psychoeducation VS Relaxation	——	——	——	——	——	——	0.80(-8.84 to 10.44)	ECCF Moderate	0.80(-8.84 to 10.44)	Moderate	NA	Very serious	Very low

a: Without considering the imprecision domain.

b: Considering the logic in the network meta-analysis.

Abbreviations: RoB = Risk of Bias, NMA = Network meta-analysis, CBT = Cognitive behavior therapy, NA = Not Applicable.

S4.5.2 Physical function when measured immediately after treatments

**Comparisons**	**Direct**	**RoB**	**Indirectness**	**Inconsistency**	**Publication Bias**	**Direct Certainty [a]**	**Indirect**	**Indirect Certainty [a]**	**NMA**	**Direct + Indirect Certainty [a]**	**Incoherence**	**Imprecision**	**NMA Certainty**
Group CBT VS Guided self-help CBT	——	——	——	——	——	——	0.46(-9.55 to 10.47)	ADDB Low	0.46(-9.55 to 10.47)	Low	NA	Serious	Very low
Group CBT VS Individual	——	——	——	——	——	——	3.56(-6.34 to 13.46)	ADDC Moderate	3.56(-6.34 to 13.46)	Moderate	NA	Serious	Very low[b]
Group CBT VS Minimal intervention	11.31(5.55 to 17.07)	Serious	Not serious	Not serious	Not serious	Moderate	NA	NA	11.33(3.24 to 19.41)	Moderate	Not serious	Very serious	Very low
Group VS Psychoeducation	——	——	——	——	——	——	5.56(-15.90 to 27.02)	ADCE Moderate	5.56(-15.90 to 27.02)	Moderate	NA	Very serious	Very low

Group VS Relaxation	——	——	——	——	——	——	25.16(5.79 to 44.53)	ADCF Moderate	25.16(5.79 to 44.53)	Moderate	NA	Serious	Very low[b]
Guided self-help CBT VS Individual CBT	7.94(-1.81 to 17.69)	Not serious	Not serious	Not serious	Not serious	High	0.97(-7.96 to 9.91)	BDDC Low	3.10(-4.35 to 10.54)	Low	Not serious	Serious	Very low
Guided self-help CBT VS Minimal intervention	9.63(0.68 to 18.58)	Not serious	Serious	Serious	Not serious	Low	16.73(1.89 to 31.57)	BCCD High	10.87(4.96 to 16.77)	Low	Not serious	Serious	Very low
Guided self-help CBT VS Psychoeducation	——	——	——	——	——	——	5.10(-15.35 to 25.54)	BCCE High	5.10(-15.35 to 25.54)	High	NA	Very serious	Very low[b]
Guided self-help CBT VS Relaxation	——	——	——	——	——	——	24.70(6.46 to 42.93)	BCCF High	24.70(6.46 to 42.93)	High	NA	Serious	Very low[b]
Individual CBT VS Minimal intervention	8.20(3.88 to 12.53)	Not serious	Not serious	Not serious	Not serious	High	1.82(-13.11 to 16.76)	CBBD Low	7.77(2.04 to 13.49)	High	Not serious	Serious	Moderate

Individual CBT VS Psychoeducation	2.00(-14.61 to 18.61)	Not serious	Not serious	Not serious	Not serious	High	NA	NA	2.00(-17.04 to 21.04)	High	Not serious	Very serious	Low
Individual CBT VS Relaxation	21.60(7.80 to 35.40)	Not serious	Not serious	Not serious	Not serious	High	NA	NA	21.60(4.95 to 38.25)	High	Not serious	Very serious	Low
Psychoeducation VS Minimal intervention	——	——	——	——	——	——	5.77(-14.12 to 25.65)	DCCE High	5.77(-14.12 to 25.65)	High	NA	Very serious	Low
Relaxation VS Minimal intervention	——	——	——	——	——	——	-13.83(-31.44 to 3.77)	DCCF High	-13.83(-31.44 to 3.77)	High	NA	Serious	Low[b]
Psychoeducation VS Relaxation	——	——	——	——	——	——	19.60(-5.69 to 44.89)	ECCF High	19.60(-5.69 to 44.89)	High	NA	Serious	Low[b]

a: Without considering the imprecision domain.

b: Considering the logic in the network meta-analysis.

Abbreviations: RoB = Risk of Bias, NMA = Network meta-analysis, CBT = Cognitive behavior therapy, NA = Not Applicable.

S4.5.3 Fatigue at the end of the follow-up

Comparisons	Direct	RoB	Indirectness	Inconsistency	Publication Bias	Direct Certainty [a]	Indirect	Indirect Certainty [a]	NMA	Direct + Indirect Certainty [a]	Incoherence	Imprecision	NMA Certainty
Group CBT VS Guided self-help CBT	——	——	——	——	——	——	-4.17(-15.55 to 7.21)	ADCB Moderate	-4.17(-15.55 to 7.21)	Moderate	NA	Very serious	Very low
Group CBT VS Individual CBT	——	——	——	——	——	——	-0.99(-7.87 to 5.89)	ADDC Moderate	-0.99(-7.87 to 5.89)	Moderate	NA	Very serious	Very low
Group CBT VS Minimal intervention	-2.49(-4.41 to -0.56)	Serious	Not serious	Not serious	Not serious	Moderate	NA	NA	-2.79(-6.97 to 1.39)	Moderate	Not serious	Very serious	Very low
Group CBT VS Psychoeducation	——	——	——	——	——	——	-2.74(-11.80 to 6.32)	ADCE Moderate	-2.74(-11.80 to 6.32)	Moderate	NA	Very serious	Very low
Group CBT VS Relaxation	——	——	——	——	——	——	-5.34(-13.74 to 3.07)	ADCF Low	-5.34(-13.74 to 3.07)	Low	NA	Very serious	Very low

Guided self-help CBT VS Individual	3.18(-4.37 to 10.73)	Serious	Not serious	Not serious	Not serious	Moderate	NA	NA	3.18(-5.88 to 12.24)	Moderate	Not serious	Very serious	Very low
Guided self-help CBT VS Minimal intervention	——	——	——	——	——	——	1.38(-9.21 to 11.97)	BCCD Moderate	1.38(-9.21 to 11.97)	Moderate	NA	Very serious	Very low
Guided self-help CBT VS Psychoeducation	——	——	——	——	——	——	1.43(-9.38 to 12.24)	BCCE Moderate	1.43(-9.38 to 12.24)	Moderate	NA	Very serious	Very low
Guided self-help CBT VS Relaxation	——	——	——	——	——	——	-1.17(-11.43 to 9.10)	BCCF Low	-1.17(-11.43 to 9.10)	Low	NA	Very serious	Very low
Individual CBT VS Minimal intervention	-1.80(-3.99 to 0.39)	Not serious	Not serious	Not serious	Not serious	High	NA	NA	-1.80(-7.27 to 3.67)	High	Not serious	Very serious	Low
Individual CBT VS Psychoeducation	-1.75(-4.85 to 1.35)	Serious	Not serious	Not serious	Not serious	Moderate	NA	NA	-1.75(-7.64 to 4.14)	Moderate	Not serious	Very serious	Very low

Individual CBT VS Relaxation	-4.93(-12.66 to 2.80)	Serious	Not serious	Serious	Not serious	Low	NA	NA	-4.35(-9.17 to 0.48)	Low	Not serious	Very serious	Very low
Psychoeducation VS Minimal intervention	——	——	——	——	——	——	-0.05(-8.09 to 7.99)	DCCE Moderate	-0.05(-8.09 to 7.99)	Moderate	NA	Very serious	Very low
Relaxation VS Minimal intervention	——	——	——	——	——	——	2.55(-4.75 to 9.84)	DCCF Low	2.55(-4.75 to 9.84)	Low	NA	Very serious	Very low
Psychoeducation VS Relaxation	——	——	——	——	——	——	-2.60(-10.21 to 5.02)	ECCF Low	-2.60(-10.21 to 5.02)	Low	NA	Very serious	Very low

a: Without considering the imprecision domain.

b: Considering the logic in the network meta-analysis.

Abbreviations: RoB = Risk of Bias, NMA = Network meta-analysis, CBT = Cognitive behavior therapy, GET = Graded exercise therapy, NA = Not Applicable.

S4.5.4 Physical function at the end of the follow-up

Comparisons	Direct	RoB	Indirectness	Inconsistency	Publication Bias	Direct Certainty [a]	Indirect	Indirect Certainty [a]	NMA	Direct + Indirect Certainty [a]	Incoherence	Imprecision	NMA Certainty
Group CBT VS Guided self-help CBT	——	——	——	——	——	——	1.39(-83.47 to 86.25)	ADDB Moderate	1.39(-83.47 to 86.25)	Moderate	NA	Very serious	Very low
Group CBT VS Individual CBT	——	——	——	——	——	——	4.90(-64.18 to 73.98)	ADDC Moderate	4.90(-64.18 to 73.98)	Moderate	NA	Very serious	Very low
Group CBT VS Minimal intervention	9.70(-6.91 to 26.31)	Serious	Not serious	Not serious	Not serious	Moderate	NA	NA	9.70(-40.29 to 59.69)	Moderate	Not serious	Very serious	Very low
Group CBT VS Psychoeducation	——	——	——	——	——	——	10.49(-74.88 to 95.86)	ADCE Moderate	10.49(-74.88 to 95.86)	Moderate	NA	Very serious	Very low
Group CBT VS Relaxation	——	——	——	——	——	——	20.51(-56.95 to 97.97)	ADCF Low	20.51(-56.95 to	Low	NA	Very serious	Very low

Guided self-help CBT VS Individual CBT	3.51(-10.80 to 17.82)	Serious	Not serious	Not serious	Not serious	Moderate	NA	NA	3.51(-45.77 to 52.79)	Moderate	Not serious	Very serious	Very low
Guided self-help CBT VS Minimal intervention	——	——	——	——	——	——	8.31(-60.26 to 76.88)	BCCD Moderate	8.31(-60.26 to 76.88)	Moderate	NA	Very serious	Very low
Guided self-help CBT VS Psychoeducation	——	——	——	——	——	——	9.10(-61.22 to 79.42)	BCCE Moderate	9.10(-61.22 to 79.42)	Moderate	NA	Very serious	Very low
Guided self-help CBT VS Relaxation	——	——	——	——	——	——	19.12(-41.34 to 79.58)	BCCF Low	19.12(-41.34 to 79.58)	Low	NA	Very serious	Very low
Individual CBT VS Minimal intervention	4.80(-2.26 to 11.86)	Not serious	Not serious	Not serious	Not serious	High	NA	NA	4.80(-42.88 to 52.48)	High	Not serious	Very serious	Low
Individual CBT VS Psychoeduca	5.59(-11.52 to 22.70)	Serious	Not serious	Not serious	Not serious	Moderate	NA	NA	5.59(-44.57 to 55.75)	Moderate	Not serious	Very serious	Very low

tion													
Individual CBT VS Relaxation	15.61(-19 .43 to 50.65)	Serious	Not serious	Serious	Not serious	Low	NA	NA	15.61(-19.43 to 50.65)	Low	Not serious	Very serious	Very low
Psychoeducation VS Minimal intervention	——	——	——	——	——	——	-0.79(-69.99 to 68.41)	DCCE Moderate	-0.79(-69.99 to 68.41)	Moderate	NA	Very serious	Very low
Relaxation VS Minimal intervention	——	——	——	——	——	——	-10.81(-69.98 to 48.36)	DCCF Low	-10.81(-69.98 to 48.36)	Low	NA	Very serious	Very low
Psychoeducation VS Relaxation	——	——	——	——	——	——	10.02(-51.17 to 71.21)	ECCF Low	10.02(-51.17 to 71.21)	Low	NA	Very serious	Very low

a: Without considering the imprecision domain.

b: Considering the logic in the network meta-analysis.

Abbreviations: RoB = Risk of Bias, NMA = Network meta-analysis, CBT = Cognitive behavior therapy, GET = Graded exercise therapy, NA = Not Applicable.

S4.5.5 Depression at the end of the follow-up

Comparisons	Direct	RoB	Indirectness	Inconsistency	Publication Bias	Direct Certainty [a]	Indirect	Indirect Certainty [a]	NMA	Direct + Indirect Certainty [a]	Incoherence	Imprecision	NMA Certainty
Group CBT VS Individual CBT	——	——	——	——	——	——	-0.11(-1.71 to 1.48)	ACCB Moderate	-0.11(-1.71 to 1.48)	Moderate	NA	Serious	Very low[b]
Group CBT VS Minimal intervention	-1.11(-2.38 to 0.16)	Serious	Not serious	Not serious	Not serious	Moderate	NA	NA	-1.11(-2.38 to 0.16)	Moderate	Not serious	Very serious	Very low
Group CBT VS Relaxation	——	——	——	——	——	——	-0.62(-2.57 to 1.34)	ACBD Moderate	-0.62(-2.57 to 1.34)	Moderate	NA	Serious	Very low[b]
Individual CBT VS Minimal intervention	-1.00(-1.96 to -0.04)	Not serious	Not serious	Not serious	Not serious	High	NA	NA	-1.00(-1.96 to -0.04）	High	Not serious	Very serious	Low

Individual CBT VS Relaxation	-0.50(-1.63 to 0.62)	Serious	Not serious	Not serious	Not serious	Moderate	NA	NA	-0.50(-1.63 to 0.62)	Moderate	Not serious	Very serious	Very low
Relaxation VS Minimal intervention	——	——	——	——	——	——	-0.50(-1.98 to 0.99)	CBBD Moderate	-0.50(-1.98 to 0.99)	Moderate	NA	Serious	Very low[b]

a: Without considering the imprecision domain.

b: Considering the logic in the network meta-analysis.

Abbreviations: RoB = Risk of Bias, NMA = Network meta-analysis, CBT = Cognitive behavior therapy, GET = Graded exercise therapy, NA = Not Applicable.

Appendix S4.6:Sensitivity analyses

S4.6.1 Sensitivity analyses by separating the minimal intervention group into waitlist and usual care.

1. Sensitivity analysis with "minimal intervention as reference" vs "with wait as reference" in fatigue.

Interventions	minimal intervention	waitlist
	Fatigue	
Individual CBT	-4.81(-6.98 to -2.65)	-4.30(-7.21 to -1.39)
Group CBT	-7.47(-10.54 to -4.39)	-7.45(-10.67 to -4.24)
Guided self-help CBT	-6.89(-9.56 to -4.22)	-6.53(-9.59 to -3.47)
Psychoeducation	-3.11(-9.57 to 3.34)	-2.60(-9.54 to 4.34)
Relaxation	-3.91(-11.69 to 3.87)	-3.40(-11.58 to 4.78)
Usual care	NA	1.18(-2.65 to 5.01)

S4.6.2 Sensitivity analyses by excluding trials involving psychoeducation or relaxation.

**Interventions**	**Main**	**Excluding trials involving psychoeducation or relaxation**
	**Fatigue**	
Individual CBT	-4.81(-6.98 to -2.65)	-4.81(-6.98 to -2.65)
Group CBT	-7.47(-10.54 to -4.39)	-7.47(-10.54 to -4.39)
Guided self-help CBT	-6.89(-9.56 to -4.22)	-6.89(-9.56 to -4.22)
Psychoeducation	-3.11(-9.57 to 3.34)	NA
Relaxation	-3.91(-11.69 to 3.87)	NA

Appendix S5: Main results at component level

Abbreviations: Ind=Individual, Gro=Group, Gui=Guided self-help, Ct=Cognitive restructuring, Ba=Behavioural activation, Ps=Psychoeducation, Ho=Homework, Pr=Problem solving, Ss=Social skills training, Re=Relaxation, Gs=Goal setting, 3w=Third-wave components.

S5.1 Network plots

5.1.1 Fatigue when measured immediately after treatments

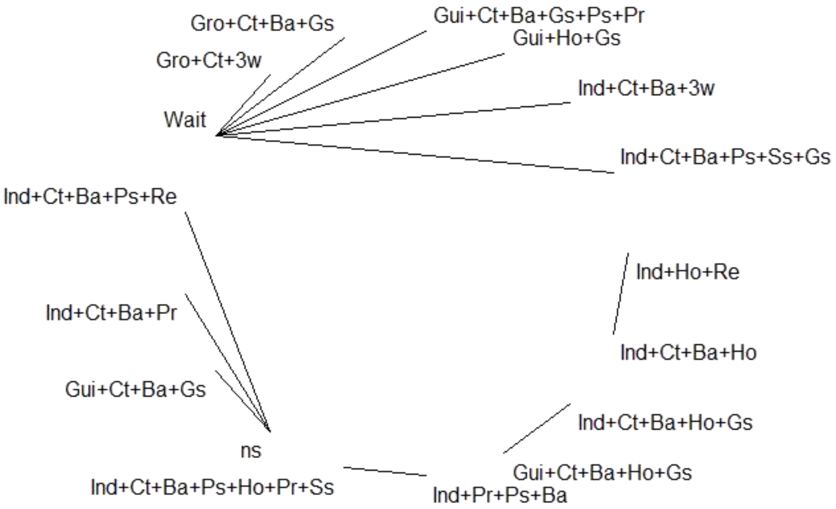

5.1.2 Physical function when measured immediately after treatments

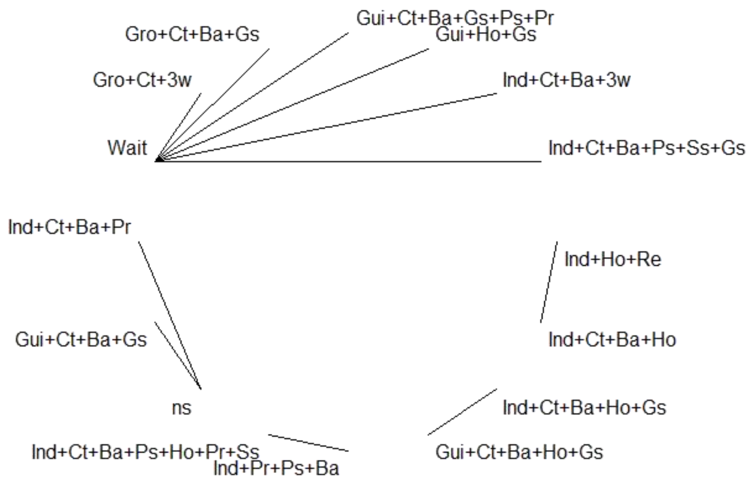

5.1.3 Fatigue at the end of the follow-up

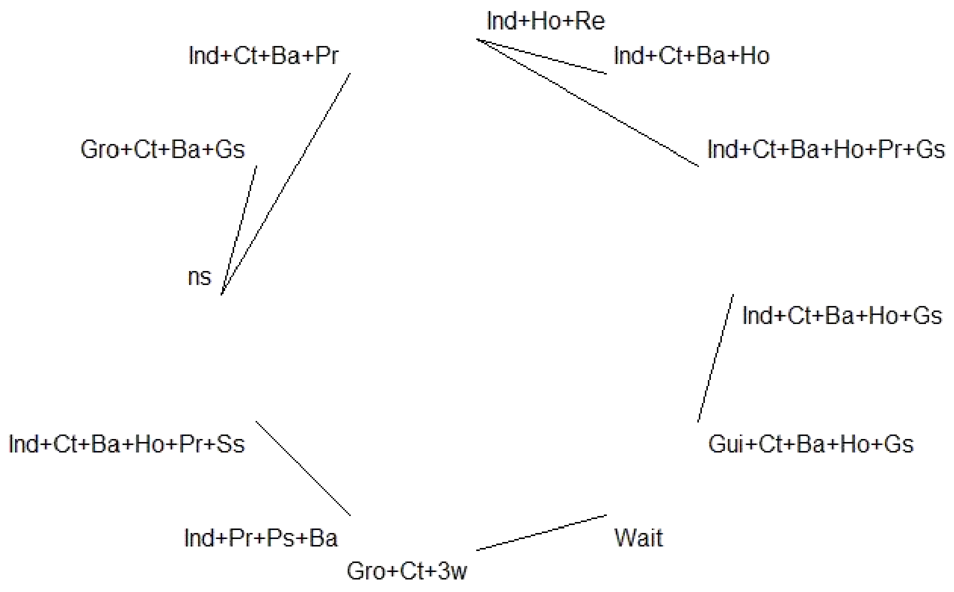

5.1.4 Physical function at the end of the follow-up

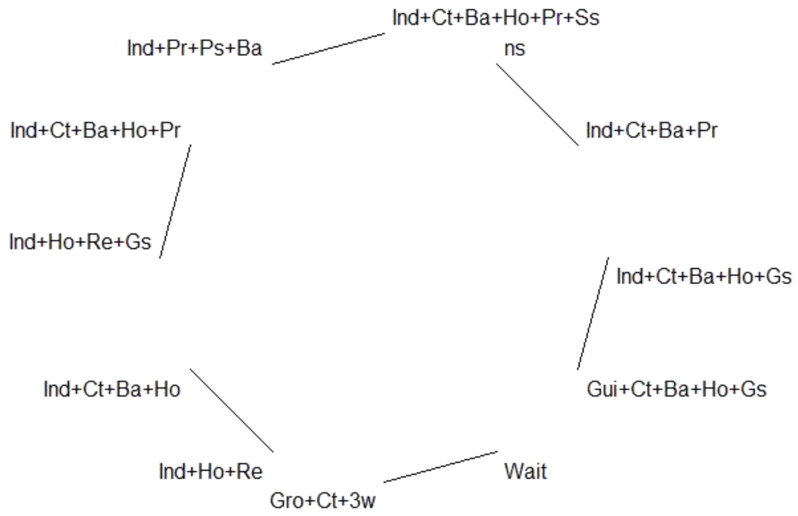

5.1.5 Depression at the end of the follow-up

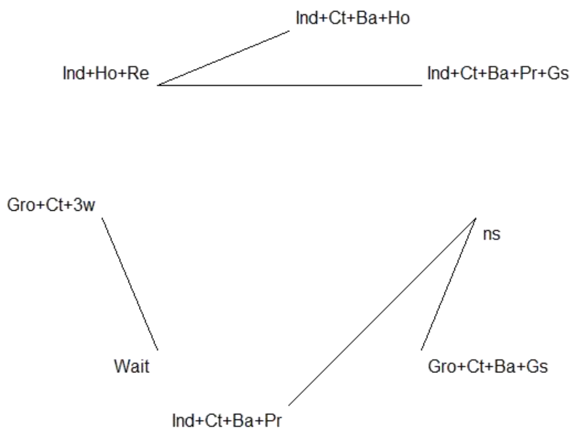

S5.2 Forest plots

5.2.1 Fatigue when measured immediately after treatments

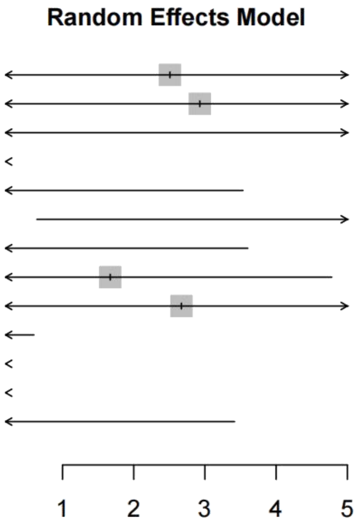

Component	MD	95%−CI	z	p−value
Ind	2.51	[−2.84; 7.86]	0.92	0.36
Gui	2.93	[−5.54; 11.39]	0.68	0.50
Gro	5.83	[−2.68; 14.34]	1.34	0.18
Ct	−4.41	[−8.83; 0.01]	−1.96	0.05
Ba	−2.27	[−8.07; 3.53]	−0.77	0.44
Ps	5.23	[0.64; 9.82]	2.23	0.03
Ho	0.04	[−3.52; 3.60]	0.02	0.98
Pr	1.67	[−1.44; 4.78]	1.05	0.29
Ss	2.67	[−3.48; 8.82]	0.85	0.39
Re	−5.78	[−12.16; 0.60]	−1.78	0.08
Gs	−8.83	[−16.92; −0.74]	−2.14	0.03
3w	−6.02	[−11.33; −0.72]	−2.22	0.03
Wait	−0.70	[−4.82; 3.42]	−0.33	0.74
ns	0.00		.	.

5.2.2 Physical function when measured immediately after treatments

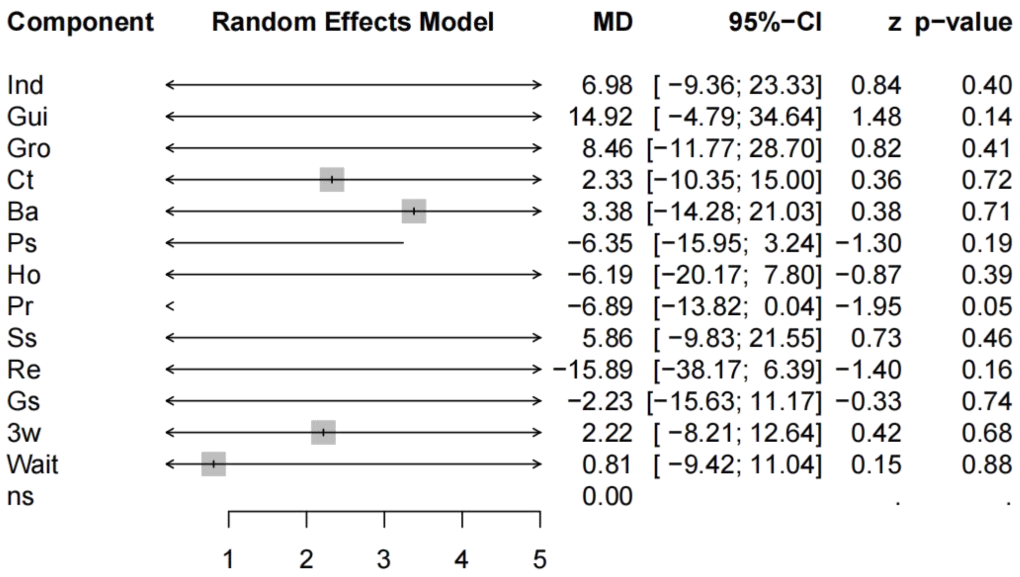

5.2.3 Fatigue at the end of the follow-up

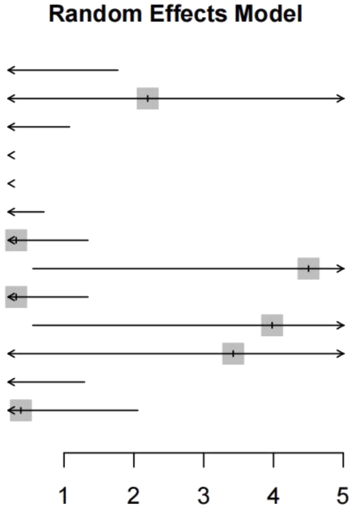

Component	MD	95%−CI	z	p−value
Ind	−0.98	[−3.74; 1.77]	−0.70	0.48
Gui	2.20	[−2.83; 7.22]	0.86	0.39
Gro	−0.24	[−1.57; 1.08]	−0.36	0.72
Ct	−2.69	[−4.32; −1.07]	−3.25	< 0.01
Ba	−2.63	[−5.33; 0.07]	−1.91	0.06
Ps	−0.31	[−1.34; 0.71]	−0.60	0.55
Ho	0.31	[−0.71; 1.34]	0.60	0.55
Pr	4.50	[0.55; 8.45]	2.23	0.03
Ss	0.31	[−0.71; 1.34]	0.60	0.55
Re	3.98	[0.55; 7.41]	2.28	0.02
Gs	3.42	[−0.30; 7.15]	1.80	0.07
3w	−0.38	[−2.06; 1.29]	−0.45	0.66
Wait	0.38	[−1.29; 2.06]	0.45	0.66
ns	0.00		.	.

5.2.4 Physical function at the end of the follow-up

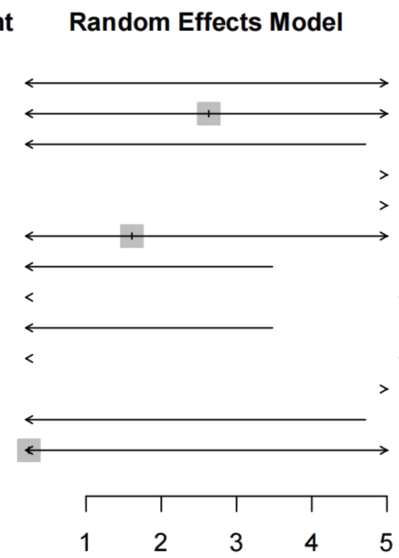

Component	MD	95%−CI	z	p−value
Ind	−0.88	[−6.90; 5.14]	−0.29	0.77
Gui	2.63	[−7.35; 12.61]	0.52	0.61
Gro	−0.24	[−5.19; 4.72]	−0.09	0.92
Ct	10.42	[5.53; 15.31]	4.17	< 0.01
Ba	12.27	[5.99; 18.54]	3.83	< 0.01
Ps	1.61	[−3.48; 6.70]	0.62	0.54
Ho	−1.61	[−6.70; 3.48]	−0.62	0.54
Pr	−17.00	[−26.90; −7.11]	−3.37	< 0.01
Ss	−1.61	[−6.70; 3.48]	−0.62	0.54
Re	−10.52	[−18.35; −2.69]	−2.63	< 0.01
Gs	18.76	[5.77; 31.74]	2.83	< 0.01
3w	−0.24	[−5.19; 4.72]	−0.09	0.92
Wait	0.24	[−4.72; 5.19]	0.09	0.92
ns	0.00		.	.

5.2.5 Depression at the end of the follow-up

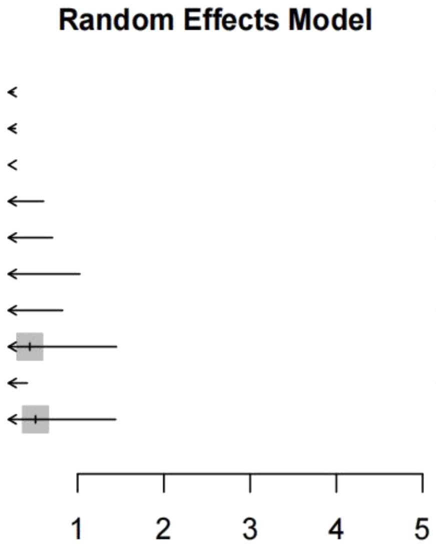

Component	MD	95%−CI	z	p−value
Ind	−0.28	[−0.83; 0.26]	−1.03	0.30
Gro	−0.43	[−1.13; 0.28]	−1.18	0.24
Ct	−0.65	[−1.18; −0.13]	−2.43	0.02
Ba	−0.14	[−0.88; 0.61]	−0.37	0.71
Ho	−0.36	[−1.43; 0.71]	−0.66	0.51
Pr	0.07	[−0.88; 1.03]	0.15	0.88
Re	−0.06	[−0.95; 0.83]	−0.13	0.89
Gs	0.45	[−0.56; 1.45]	0.87	0.39
3w	−0.51	[−1.44; 0.42]	−1.08	0.28
Wait	0.51	[−0.42; 1.44]	1.08	0.28
ns	0.00		.	.

S5.3 Sensitivity analyses

	Beneficial	Harmful
P＞0.05		
0.01＜P≤0.05		
P≤0.01		

	S1	S2	S3	S4
	Excluding high risk of bias	Excluding delivery method components	Excluding small trials	Excluding inactive arms
	**iMD (95% CI)**	**iMD (95% CI)**	**iMD (95% CI)**	**iMD (95% CI)**
Individual	0.60(-2.33 to 3.53)	-	-0.21(-2.27 to 1.86)	-2.35(-5.76 to 1.05)
Group	6.17(-2.81 to 15.15)	-	1.73(-2.62 to 6.08)	-
Guided self-help	1.02(-6.71 to 8.75)	-	0.21(-6.17 to 6.60)	-1.93(-5.24 to 1.37)
Cognitive restructuring	-4.93(-9.24 to -0.61)	-1.24(-5.82 to 3.33)	-5.74(-10.76 to -0.72)	-1.32(-2.92 to 0.28)
Behavioural activation	-0.02(-4.18 to 4.15)	-3.19(-7.88 to 1.50)	1.33(-4.45 to 7.12)	-1.13(-3.20 to 0.94)
Psychoeducation	3.66(0.85 to 6.47)	2.47(-0.65 to 5.59)	3.40(0.17 to 6.64)	1.62(-0.61 to 3.84)
Homework	0.38(-3.21 to 3.98)	-1.07(-4.17 to 2.04)	0.93(-2.58 to 4.43)	-0.19(-2.01 to 1.63)
Problem solving	1.84(-1.15 to 4.84)	1.72(-0.85 to 4.28)	2.11(-0.69 to 4.92)	2.30(-1.01 to 5.60)
Social skills training	2.84(-3.23 to 8.91)	1.72(-1.67 to 5.10)	3.11(-3.04 to 9.27)	-0.19(-2.01 to 1.63)
Relaxation	-4.04(-8.15 to 0.07)	-2.80(-7.04 to 1.44)	-3.51(-7.66 to 0.65)	-1.54(-4.76 to 1.68)
Goal setting	-8.66(-16.79 to -0.53)	-8.15(-12.02 to -4.28)	-8.39(-16.30 to -0.47)	-8.20(-13.15 to -3.25)
Third-wave components	-7.24(-13.63 to -0.86)	-7.23(-13.49 to -0.96)	-6.96(-13.19 to -0.73)	-
Waiting component	-2.09(-4.91 to 0.72)	-3.78(-7.52 to -0.03)	-2.08(-5.57 to 1.40)	-

S5.4 Subgroup analyses

	Gender		Age	
	**Female>80%**	**Female≤80%**	**age≤18**	**age＞18**
	k=4	k=8	k=4	k=8
	**iMD (95% CI)**	**iMD (95% CI)**	**iMD (95% CI)**	**iMD (95% CI)**
Individual	-0.50(-2.75 to 1.75)	-0.88(-3.45 to 1.69)	-0.37(-1.46 to 0.72)	-0.28(-2.28 to 1.71)
Group	0.90(-1.51 to 3.30)	-2.44(-4.96 to 0.08)	-	-2.46(-4.39 to -0.53)
Guided self-help	-3.69(-4.92 to -2.47)	-0.46(-4.55 to 3.63)	-3.11(-4.29 to -1.92)	0.14(-4.48 to 4.76)
Cognitive restructuring	-3.30(-4.27 to -2.32)	-1.18(-2.84 to 0.48)	-2.89(-4.09 to -1.68)	-0.58(-2.51 to 1.34)
Behavioural activation	-4.19(-6.23 to -2.16)	-1.61(-4.31 to 1.08)	-3.48(-4.43 to -2.53)	-1.49(-3.65 to 0.67)
Psychoeducation	2.29(0.87 to 3.72)	0.84(-2.34 to 4.03)	1.01(-0.02 to 2.04)	1.54(-1.38 to 4.47)
Homework	-	-0.96(-2.59 to 0.67)	0.59(-1.06 to 2.25)	-0.85(-2.59 to 0.88)
Problem solving	-	1.17(-2.03 to 4.38)	-	-0.15(-2.24 to 1.95)
Social skills training	2.29(0.87 to 3.72)	0.44(-2.29 to 3.16)	0.59(-1.06 to 2.25)	0.85(-4.41 to 6.12)
Relaxation	-	-1.89(-6.27 to 2.49)	1.01(-0.02 to 2.04)	-1.17(-6.66 to 4.32)
Goal setting	-1.40(-3.07 to 0.27)	-1.87(-3.89 to 0.15)	-3.11(-4.29 to -1.92)	-2.68(-4.97 to -0.38)
Third-wave components	-1.90(-3.48 to -0.32)	-	-1.38(-3.18 to 0.42)	0.91(-1.03 to 2.85)
Waiting component	-0.40(-1.36 to 0.57)	1.87(-0.15 to 3.89)	1.38(-0.42 to 3.18)	1.77(-0.52 to 4.06)

S5.5 The additive model evaluating delivery–content combinations

Beneficial components of CBT treatment	Delivery Formats of CBT	delivery–content combinations iMD (95% CI)
Ct+3w+Gs	Gui	-16.33(-26.68 to -5.99)
	Gro	-13.43(-25.23 to -1.63)
	Ind	-16.75(-31.94 to -1.57)
